# Genome-Wide Association Study on Resistance to Stalk Rot Diseases in Grain Sorghum

**DOI:** 10.1534/g3.114.016394

**Published:** 2015-04-16

**Authors:** Adedayo Adeyanju, Christopher Little, Jianming Yu, Tesfaye Tesso

**Affiliations:** * Department of Agronomy, Kansas State University, Manhattan, Kansas 66506; † Department of Plant Pathology, Kansas State University, Manhattan, Kansas 66506,; ‡ Department of Agronomy, Iowa State University, Ames, Iowa 50011

**Keywords:** sorghum, *Fusarium thapsinum*, *Macrophomina phaseolina*, stalk rots, association mapping, sorghum diversity panel

## Abstract

Stalk rots are important biotic constraints to sorghum production worldwide. Several pathogens may be associated with the disease, but *Macrophomina phaseolina* and *Fusarium thapsinum* are recognized as the major causal organisms. The diseases become more aggressive when drought and high-temperature stress occur during grain filling. Progress in genetic improvement efforts has been slow due to lack of effective phenotyping protocol and the strong environmental effect on disease incidence and severity. Deployment of modern molecular tools is expected to accelerate efforts to develop resistant hybrids. This study was aimed at identifying genomic regions associated with resistance to both causal organisms. A sorghum diversity panel consisting of 300 genotypes assembled from different parts of the world was evaluated for response to infection by both pathogens. Community resources of 79,132 single nucleotide polymorphic (SNP) markers developed on the panel were used in association studies using a multi-locus mixed model to map loci associated with stalk rot resistance. Adequate genetic variation was observed for resistance to both pathogens. Structure analysis grouped the genotypes into five subpopulations primarily based on the racial category of the genotypes. Fourteen loci and a set of candidate genes appear to be involved in connected functions controlling plant defense response. However, each associated SNP had relatively small effect on the traits, accounting for 19–30% of phenotypic variation. Linkage disequilibrium analyses suggest that significant SNPs are genetically independent. Estimation of frequencies of associated alleles revealed that durra and caudatum subpopulations were enriched for resistant alleles, but the results suggest complex molecular mechanisms underlying resistance to both pathogens.

Sorghum is the world’s fifth most important cereal crop. It is a staple food for more than half a billion people in the world, 60% of whom live in Africa. As a C4 species, sorghum has very high yield potential comparable with maize and rice. However, numerous biotic and abiotic stresses pose formidable challenges to the realization of this potential. In addition to various cropping systems and management practices, host plant resistance and/or tolerance to these stresses have been widely recognized as the most viable approach to enhancing productivity under these conditions (Waniska *et al.* 2001).

Stalk rots are among the most important diseases of sorghum worldwide. Of the many types of stalk rot diseases, *Fusarium* stalk rot caused by *Fusarium spps* and charcoal rot caused by *Macrophomina phaseolina* are the most important. The diseases cause significant damage to root and stalk tissues of infected plants, interfering with absorption and translocation of minerals and water. In severe cases, the disease results in complete stalk collapse, leading to lodging. Recent studies have indicated increased incidence of stalk rot worldwide (Mahmoud and Budak 2011; Khangura and Aberra 2009), highlighting the growing importance of the disease. *M. phaseolina*, a soil-borne necrotrophic fungus, is the most aggressive stalk rot pathogen in sorghum (Crasta *et al.* 1999). It enters host cells through the roots and continues to the base of the plant, resulting in disintegration of the pith cells and eventually leading to premature senescence and lodging. *Fusarium* spp. also have a similar mode of infection, but they tend to be more problematic under temperate-type environments, unlike *M. phaseolina*, which prefers dry and high-temperature conditions (Bramel-Cox *et al.* 1988; Klittich *et al.* 1997; Marasas *et al.* 2001; Leslie 1995; Leslie *et al.* 1990).

Yield reductions associated with stalk rot diseases are common in almost all sorghum-producing regions of the world, including Africa (Frowd 1980; Omar 1985; Hulluka and Esele 1992), India (Khune 1984; Seetharama *et al.* 1987), Australia (Trimboli and Burgess 1982), and the United States (Jardine and Leslie 1992; Reed *et al.* 1983). The extent of the reduction, however, may vary from region to region depending on disease severity.

Previous studies have reported significant genetic variability for reaction to stalk rot pathogens, identified sources of resistance, and determined the mode of inheritance of the traits for both *Fusarium* stalk rot and charcoal rot (Bramel-Cox *et al.* 1988; Sahib *et al.* 1990; Pecina-Quintero *et al.* 1999; Tesso *et al.* 2005). However, large-scale phenotyping for stalk rot resistance in the breeding nursery remains difficult because of the tedious nature of the manual inoculation and disease scoring procedure. Nevertheless, because many of the resistance sources were identified in stay-green backgrounds, and because previous studies showed strong association between charcoal rot resistance and stay-green–mediated postflowering drought tolerance, much of the breeding efforts to improve the traits have mainly focused on indirect selection for the stay-green trait. Although this approach has worked well, it obviously leaves out potential resistance sources that may be available in nonstay-green backgrounds. Moreover, stay-green is just one of the many mechanisms for drought tolerance in plants, and that alone may not explain the complex response of genotypes to drought stress and also infection by stalk rot pathogens. Additional sources of resistance/tolerance and new tools and techniques for tracking the associated traits are needed to make meaningful progress in breeding for these diseases.

Genome-wide association mapping has arisen as a powerful tool for high-resolution mapping of loci underlying quantitative traits. Because it takes advantage of accumulated historic recombination events in natural populations, it holds promise for identifying causative polymorphism of complex traits (Stich *et al.* 2008). But false discovery attributed to spurious associations caused by population stratification and unequal relatedness need to be accounted for (Abiola *et al.* 2003). This concern is usually addressed using general linear model (GLM)-based methods such as structured association (Pritchard *et al.* 2000), genomic control (Devlin and Roeder 1999), and family-based tests of association (Abecasis *et al.* 2000). Association analysis is cheaper and faster because it utilizes existing populations such as landraces, modern cultivars, and advanced breeding lines to detect potential associations between molecular markers and traits of interest (Zhu *et al.* 2008). It has been successfully applied to identify marker-trait associations in different crops (Beattie *et al.* 2010; Kump *et al.* 2011; Roy *et al.* 2010; Wang *et al.* 2012a; Yu *et al.* 2006; Zhu *et al.* 2008; Gupta *et al.* 2005). In sorghum, it has been used to map agronomic characteristics and grain quality traits (Sukumaran *et al.* 2012; Figueiredo *et al.* 2010), plant height and maturity (Upadhyaya *et al.* 2012; Brown *et al.* 2008), agro-climatic traits (Morris *et al.* 2013), biomass, grain yield, and saccharification (Yi-Hong 2012), and days to flowering and yield components (Shehzad *et al.* 2009).

In the present study, sorghum association panel consisting of 300 accessions previously characterized with 265,487 SNPs were investigated. The panel was phenotyped for resistance to *F. thapsinum*–induced and *M. phaseolina*–induced stalk rot diseases. The objective was to determine marker-trait relationship and identify SNP markers significantly associated with resistance to these diseases.

## Materials and Methods

### Genetic materials and field management

A sorghum association panel consisting of 300 genotypes (251 converted tropical sorghums and 49 breeding lines from the United States) was used in this study. The genotypes were planted in three environments in a randomized complete block design and replicated twice. The tests were conducted at the Kansas State University (KSU) Agronomy Research farm at Ashland Bottoms near Manhattan, Kansas, in the 2011 and 2012 seasons, and at the KSU North East Agricultural Experiment Station near Ottawa, Kansas, in the 2012 season. Soil at Manhattan (Ashland Bottoms) was a well-drained Smolan Silty Clay Loam, whereas the Ottawa soil is Harney Silt Loam. Plots were 6-m-long single rows spaced 0.75 m apart with a 0.6-m alley at the end of each plot. Three grams of seeds of each entry were drilled into the plots after treatment with herbicide safener (Maxim 4FS, Apron XL, Concept III, and colorant) and the seedlings were later thinned to approximately 40 plants per plot. Fertilizer phosphorous [di-ammonium phosphate (DAP)] and nitrogen (urea) were applied at the rate of 34 kg P_2_O_5_ ha^−1^ and 90 kg N ha^−1^, respectively, at Manhattan, and 25 kg P_2_O_5_ ha^−1^ and 90 kg N ha^−1^, respectively, at Ottawa. Weeds were controlled with preplant application of 0.24 L ha^−1^ Dual plus 0.68 kg ha^−1^ Atrazine. Postemergence weeds were removed manually. At flowering, six random plants in each plot were tagged with two distinct tagging tapes, blue and red, three plants for each color.

### Inoculum preparation and inoculation

Two fungal pathogens, *Fusarium thapsinum* and *Macrophomina phaseolina*, were selected for this study. Previous reports indicate that these two organisms are more virulent than other stalk-associated fungi and are most frequently associated with stalk rot diseases in sorghum (Tesso *et al.* 2010).

For *F. thapsinum*, liquid inoculum suspensions were initiated in potato dextrose broth (DIFCO, Detroit, MI) from a pure culture of the pathogen provided by Dr. Chris Little of the Department of Plant Pathology at KSU. The suspensions were incubated at room temperature on a rotary shaker until conidia were produced and then strained through four layers of cheesecloth to separate the conidia from the mycelial mass. The number of conidia in the suspension was determined by counting the spores under a microscope using a hemacytometer and the concentration was adjusted to 5 × 10^4^ conidia ml^−1^ by diluting with 10 mM (pH 7.2) Phosphate-buffered saline and kept on ice until inoculation. At 14 d after flowering, approximately 1 ml of the suspension was injected into the basal stalks of the three red-tagged plants in each plot. An Idico filler-plug gun (Forestry Suppliers, Inc., Jackson, MS) equipped with a stainless steel needle similar to that described by Toman and White (1993) was used to deliver the conidial suspension.

**Figure 1:**
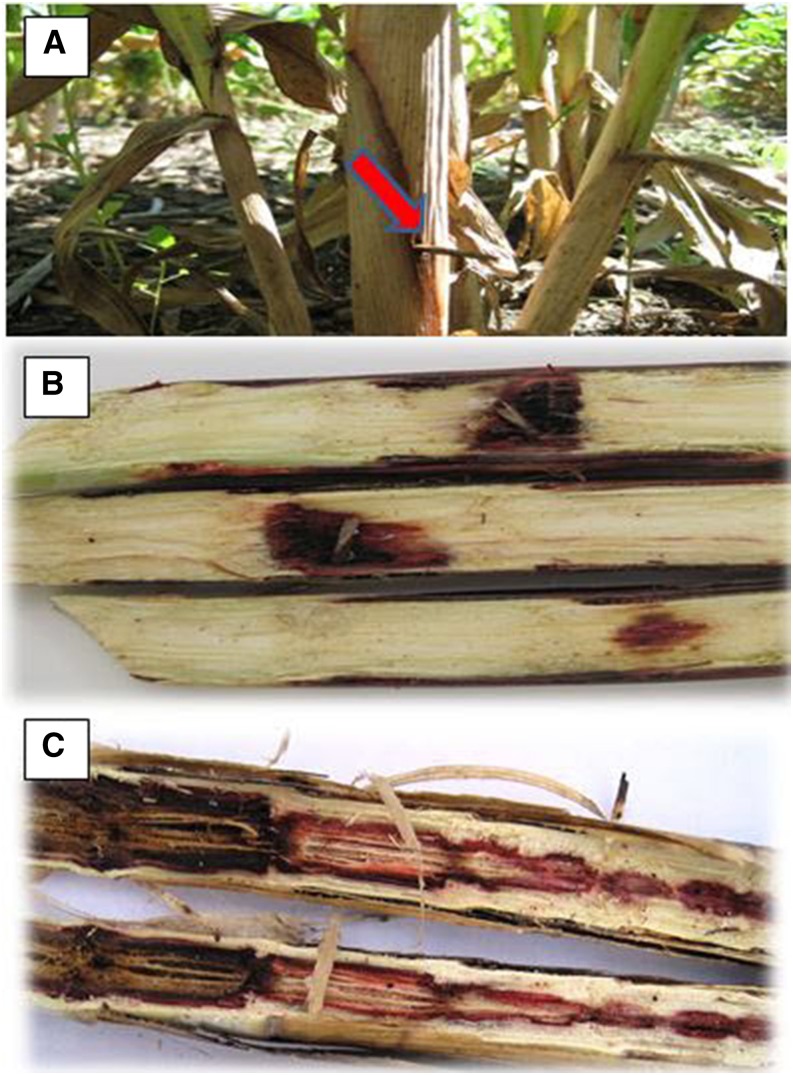
Field procedure for administering the treatments and scoring phenotypic data. Artificial inoculation with *Macrophomina phaseolina* using the toothpick method (A). Longitudinally split stalk of resistant (B) and susceptible (C) genotypes showing contrasting tissue lesion and stalk disintegration by the pathogen.

A pure culture of *M. phaseolina* was also provided by Dr. Chris Little. The pathogen was then subcultured into several fresh potato dextrose agar plates into which sterile toothpicks were inserted. The culture was incubated at 30° for 2 wk and the infested toothpicks were directly used for inoculation. Again, on d 14 after flowering, the remaining three blue-tagged plants in each plot were inoculated by inserting infested toothpicks into the basal stalks (approximately 10 cm above soil surface) through holes made with a sterilized needle (Figure 1A).

### Data collection

#### Phenotypic data:

Four weeks after inoculation, the plants were cut at the crown and the stalks were split longitudinally through the points of inoculation (Figure 1, B and C). Disease severity was scored by measuring the length (mm) of the visible lesion as follows. Total lesion length (TLL) was obtained by measuring the entire length of the visibly discolored region of the pith. Major lesion length (MLL) was estimated as the length of a continuous necrotic area that covered at least half of the stalk diameter. Relative total lesion length (RTLL) and relative major lesion length (RMLL) were estimated as the ratio of the TLL and MLL to plant height, respectively. Plant height (cm) was measured as the average length of three plants in a plot measured from the tip of the panicle to the base of the stalk. Days to flowering was recorded as the number of days taken from planting until 50% of the plants in a plot reached half-bloom stage.

#### Genotypic data:

The genotype data were a community resource of 265,487 SNPs (Morris *et al.* 2013) generated on the entire sorghum association panel. To avoid potential false findings, only 79,132 SNP markers that were mapped to a defined single location in the sorghum genome and that had <20% missing data and minor allele frequency (MAF) >0.05 were selected and used in the association analysis.

### Statistical analysis

#### Estimation of least-square means and repeatability:

Genotype means adjusted for environmental effects were obtained by using the method described by Zila *et al.* (2013). Data from each environment were first analyzed individually and combined analysis was performed on data pooled across environments. A mixed linear model was used for analyzing the data with genotypes treated as fixed effect, days to flowering treated as a fixed linear covariate, and year, genotype × year interaction, and replication within years treated as random effects. In the across environment combined analysis, genotypes were considered as fixed effect, days to flowering was considered as a fixed covariate nested within environment, and environment, genotype × environment interaction (G × E), and replication within environment were treated as random effects. All analyses were weighted by the number of stalks scored within each plot and utilized a heterogeneous error variance structure. In both experiments, larger predicted stalk rot trait values were associated with larger residuals, so a natural logarithmic transformation of raw stalk rot scores (which largely eliminated the relationship between residual variance and predicted values) was used for all analyses. Least square means were estimated for genotypes within each environment and across environments using ASReml version 3 (Gilmour *et al.* 2009).

A second analysis in which genotypes were treated as random effects was performed for the purpose of estimating repeatability. The same models described above were used to obtain estimates of genetic variance.

Repeatability on genotype mean-basis was estimated as,

$${\hat{H}}_{c}=1-\frac{\sigma_{PPE}^{2}}{2{\hat{\sigma }}_{G}^{2}}$$,

where $\sigma_{PPE}^{2}\;$ is the average prediction error variance for all pairwise comparisons of lines and ${\hat{\sigma }}_{G}^{2}$ is the estimated genetic variance (Cullis *et al.* 2006).

Repeatability was estimated for each environment individually, across years for the Manhattan environments, and for the combined data across all three environments. The model used to estimate repeatability on genotype mean basis in the combined data was modified by nesting the random genotype effect within environment and by modeling the genotype–environment effect (G) matrix as unstructured, thereby allowing estimation of unique genetic variance within each environment and a unique genetic correlation between each pair of environments. The same models used to compute repeatability for all stalk rot response parameters were used to estimate repeatability for days to flowering and plant height. Genetic correlations among stalk rot traits were estimated from least square means obtained from the combined analysis using a multivariate model in ASReml version 3 (Gilmour *et al.* 2009).

#### Population structure and kinship analysis:

Population structure was investigated to define suitable covariates for association genetic models. A Bayesian model-based clustering method implemented in STRUCTURE software (Pritchard *et al.* 2000) was used to detect population structure and to assign individuals to subpopulations. This analysis requires selectively neutral polymorphic unlinked markers, and those markers were selected using PLINK software to generate a pruned subset of SNPs that are in approximate linkage equilibrium with each other. The final SNP marker dataset used to analyze the population structure thus contained 25,190 markers from 10 linkage groups. An initial cluster of 2 through 10 were tested with 10 replications for each value of putative *k* clusters, and the log probability of data was estimated using the admixture model with correlated allele frequency. The initial 10^5^ steps were discarded as burn-in to allow the run parameters to attain convergence, after which data were collected for an additional 5 × 10^5^ steps. Using stability of the rate of change of log likelihood across grouping patterns within the 10 runs, the optimal (*k*) was observed between *k* = 4 and 5. The optimal *k* was determined based on the likelihood plot of models and stability of grouping patterns across 20 runs. Subsequently, we tested clusters 4 and 5 using an initial burn-in of 150,000, after which data were collected for an additional 350,000 steps with 20 replications for each cluster. Further, the membership coefficients for the most optimal number of clusters based on this analysis were permuted using the Greedy algorithm to match the various replicates for that value of *k* as closely as possible using CLUMPP software (Jakobsson and Rosenberg 2007). Finally, the membership assignments of individuals in various clusters were visualized using a plotting function in DISTRUCT software (Rosenberg 2004). Probabilities of subpopulation membership coefficients (*Qk*) were used for assigning lines to subpopulations. Lines with the highest membership probability (*Qk* less than 0.8 for all *k*) were considered to result from admixture and, hence, classified as “mixed.” Kinship (K) was estimated using the software SPAGeDi 1.4 (Hardy and Vekemans 2002) using the method of Ritland (1996).

### Association analysis

The least square means of genotypes for the various disease parameters were used as the input phenotype. The genome-wide association study (GWAS) was conducted on the phenotype and 79,132 genome-wide SNP markers data using univariate unified mixed linear model (Yu *et al.* 2006), so it was not necessary to re-compute variance components. The optimum compression mixed linear model and P3D options (*i.e.*, population parameters previously determined) that increase computational speed and statistical power were implemented by clustering individuals in groups in the Genome Association and Prediction Integrated Tool package (GAPIT) (Zhang *et al.* 2010; Lipka *et al.* 2012). To control for population structure and familial relatedness, the mixed model included principal components (Price *et al.* 2006) and a kinship (co-ancestry) matrix (Ritland 1996). An *R^2^* statistic was used to assess the amount of phenotypic variation explained by the model. The procedure by Benjamini and Hochberg (1995) was used to control for the multiple testing problem at false discovery rates (FDRs) of 5%.

In addition to the unified mixed linear model, a multi-locus mixed model (MLMM) (Segura *et al.* 2012) was used to dissect complex association signals that involved major effect loci. The MLMM uses stepwise mixed-model regression with forward inclusion and backward elimination, thus allowing for a more exhaustive exploration of the model space. In contrast to the unified mixed model with P3D, the MLMM re-estimates the genetic and error variance components at each step of the regression. Specifically, all SNPs on a chromosome with a major effect locus were considered for inclusion into the final model. The optimal model was selected using the extended Bayesian information criterion (Chen and Chen 2008). We then reanalyzed GWAS for each trait with MLMM-identified SNPs included as covariates in the unified mixed linear model for better control of major effect loci. Linkage disequilibrium analyses of the significantly associated SNPs were performed with HAPLOVIEW v.4.2 (Barrett *et al.* 2005), and the candidate genes located within or adjacent to associated SNPs were identified using the sorghum genome browser (Goodstein *et al.* 2012).

#### Allele frequency analysis:

Population structure analysis sorted the genotypes into four subpopulations that presumably reflected racial category of the accessions. Genotypes with mixed ancestry were dropped from the analysis. The frequency of alleles associated with low stalk rot infection was estimated. Based on the results of the GWAS analyses, the frequencies of alleles that reduced disease severity at significant SNPs were estimated within each subpopulation using the FREQ package in R software version 3.0.2 (R CORE TEAM 2013). At each SNP locus, a Fisher’s exact test using the EXACT package in R software version 3.0.2 (R CORE TEAM 2013) was used to test the null hypothesis that frequency of the allele conferring increased disease resistance was the same across all four subpopulations.

## Results

### Response of genotypes to infection by *M. phaseolina* and *F. thapsinum*

Genotypic response to stalk rot infection caused by *F. thapsinum* and *M. phaseolina* was assessed in a sorghum association panel consisting of 300 genotypes. Mean disease rating among the genotypes was markedly variable in all test environments for both pathogen species. For *F. thapsinum*, mean disease score for TLL ranged from 13.3 to 346.7 mm at Manhattan and from 16.7 to 143.4 mm at Ottawa with location means of 40.9 and 55.6 mm, respectively (Table 1). The highest disease scores were 26- and 9-times as high as the least scores at the Manhattan and Ottawa locations, respectively. The trend was similar for MLL as well, with location means of 47.7 and 37.4 mm and ranges of 20.2 to 104 mm and 13.4 to 105.8 mm for Manhattan and Ottawa, respectively. Results for *M. phaseolina* were similar to *F. thapsinum*, except that the ratings were slightly lower at Manhattan, with disease scores of 46.2 and 40.7 mm for TLL and MLL compared to 55.6 and 47.7 mm for *F. thapsinum* at the same location. However, the result was the opposite at Ottawa, where disease ratings for *M. phaseolina* were slightly higher than that of *F. thapsinum* (Table 1). For both pathogens, mean disease ratings were slightly higher at the Manhattan location.

**Table 1:** Means and repeatability estimates for stalk rot traits

Environment	Ottawa 2012			Manhattan 2011 and 2012 Combined			Overall Combined		
Traits	Mean*^a^*	Range	${\hat{H}}_{c}$	Mean*^a^*	Range	${\hat{H}}_{c}$	Mean*^a^*	Range	${\hat{H}}_{c}$
***F. thapsinum***									
Total lesion length (TLL)	4.09	1.27–11.82	0.79	5.56	1.83–16.37	0.95	5.06	2.20–12.36	0.69
Major lesion length (MLL)	3.74	1.34–10.58	0.82	4.77	2.02–10.40	0.96	4.43	2.07–10.32	0.75
Relative TLL	0.05	0.02–0.14	0.71	0.05	0.02–0.13	0.97	0.05	0.02–0.12	0.64
Relative MLL	0.05	0.02–0.14	0.73	0.04	0.02–0.11	0.97	0.04	0.02–0.10	0.66
***M. Phaseolina***									
Total lesion length (TLL)	4.19	1.17–26.31	0.93	4.62	2.18–12.26	0.93	4.49	1.97–15.67	0.78
Major lesion length (MLL)	3.85	1.1721.30	0.93	4.07	1.85–12.71	0.94	4.00	1.94–11.24	0.80
Relative TLL	0.05	0.02–0.27	0.89	0.04	0.02–0.11	0.96	0.04	0.02–0.15	0.71
Relative MLL	0.05	0.02–0.22	0.87	0.04	0.02–0.13	0.97	0.04	0.02–0.10	0.73
Plant height	84.9	27.5–160	0.75	113.5	63.2–228.3	0.87	104	62.5–190.8	0.78
Days to flowering	70	53–78	0.90	63	48–77	0.68	65.4	50–80	0.53

^*a*^ Means are reported as the average of the least-square means calculated within and across environments.

In the combined analysis, disease ratings for *F. thapsinum* were 50.6 and 44.3 mm for TLL and MLL, respectively, as compared to TLL of 44.9 and MLL of 40.0 mm for *M. phaseolina*. Also, there was significant genotype × environment interaction for all disease parameters. Estimates of genotypic covariance from the mixed model analysis for each trait and for each pair of environments showed that the two Manhattan environments had a much stronger genotypic correlation for all parameters (Table 2) than did any other pair of environments. Thus, there was little G × E interaction detected in the analysis across the two Manhattan environments. However, pair-wise genotypic correlations were much lower between Manhattan and Ottawa environments for all traits (Table 2), indicating marked variation in genotypic response between these two environments. This was also evident from the significant G × E interaction in the combined analysis.

**Table 2:** Genotypic correlations from the combined analysis of sorghum diversity panel evaluated in three environments

	Disease Traits*^a^*	*M. phaseolina*				*F. thapsinum*				Days to Flowering	Plant Height
		TLL	MLL	RTLL	RMLL	TLL	MLL	RTLL	RMLL		
*M. phaseolina*	TLL	**0.16**	0.14	0.10	0.86	0.11	0.11	0.07	0.60	−0.31	0.65
	MLL	0.99	**0.13**	0.84	0.85	0.97	0.10	0.63	0.62	−0.22	0.69
	RTLL	0.11	0.09	**0.10**	0.98	0.08	0.08	0.07	0.82	−0.41	0.22
	RMLL	0.09	0.08	0.08	**0.07**	0.07	0.07	0.06	0.06	−0.31	0.21
*F. thapsinum*	TLL	0.94	0.10	0.86	0.90	**0.09**	0.09	0.06	0.79	−0.10	0.57
	MLL	0.90	0.94	0.80	0.85	0.99	**0.09**	0.74	0.78	−0.03	0.59
	RTLL	0.06	0.06	0.86	0.93	0.06	0.06	**0.06**	0.99	−0.14	−0.09
	RMLL	0.06	0.06	0.06	0.89	0.06	0.06	0.06	**0.06**	−0.06	−0.04
Days to flowering		−0.57	−0.36	−0.58	−0.38	−0.13	−0.05	−0.16	−0.07	**21.04**	0.99
Plant height		5.58	5.36	1.45	1.22	3.65	3.92	−0.47	−0.19	0.01	**467.40**

The bold diagonal numbers are an estimate of genetic variance for each trait. Estimates of covariance between pairs of traits are shown below the diagonal, and genetic correlations between traits are shown above the diagonal. TLL, total lesion length; MLL, major lesion length; RTLL, relative total lesion length; RMLL, relative major lesion length.

Genetic correlations were estimated between all disease-related parameters, plant height, and days to flowering (Table 2). MLL and TLL were highly correlated in *M. phaseolina* and in *F. thapsinum*. In both *M. phaseolina* and *F. thapsinum*, relative total lesion length (RTLL) was significantly correlated with MLL and relative major lesion length (RMLL), but not with TLL. However, RMLL was significantly correlated with TLL as well as MLL for both pathogens. Moreover, TLL and RTLL in *F. thapsinum* were significantly and positively correlated with MLL in *M. phaseolina*, but not with all other disease traits. RMLL was also not correlated with any disease trait in *M. phaseolina*. However, RMLL in *F. thapsinum* was significantly and positively correlated with all disease parameters in *M. phaseolina*, except RMLL, for that pathogen. Correlations between plant height and stalk rot traits were contradicting. Plant height was significantly correlated with TLL and MLL both in *M. phaseolina*–based and *F. thapsinum*–based scores but not with RTLL and MTLL for both pathogens. Correlation between days to flowering and disease traits was negative and low or not significant for both pathogens, but there was a strong positive correlation between days to flowering and plant height.

### Population structure

**Figure 2:**
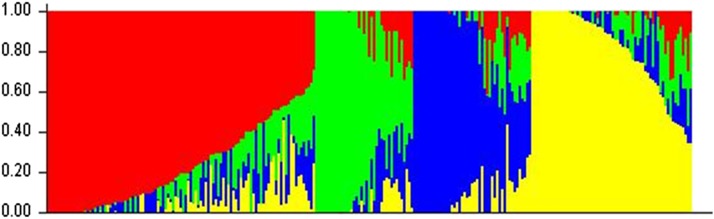
Population structure plot for the 257 entries based on 25,000 SNPs. The red, green, blue, and yellow bars correspond to the caudatum, kafir, guinea, and durra races, respectively. Vertical and horizontal axes represent the membership coefficient and genotypes, respectively.

Previous studies of these populations have assigned the genotypes to five subpopulations based on a probability of membership *P* ≥ 0.5. This subgrouping was largely based on botanical races of the genotypes. In the current study, a few of these genotypes were reassigned to the admixture group, perhaps due to a more stringent threshold for probability of membership, which is *P* ≥ 0.8. The majority of the bicolor group was reassigned to the admixed group. A majority of the genotypes that were reassigned from one of the population groups to the mixed group in the current analysis had probability of membership (*P* = 0.60–0.79) in their previously assigned group (Figure 2, Supporting Information, Table S3). This indicates that if the arbitrary threshold of *P* ≥ 0.5 were used, then the population subgrouping would have become similar to the previous grouping.

### Genome-wide association analysis

**Figure 3:**
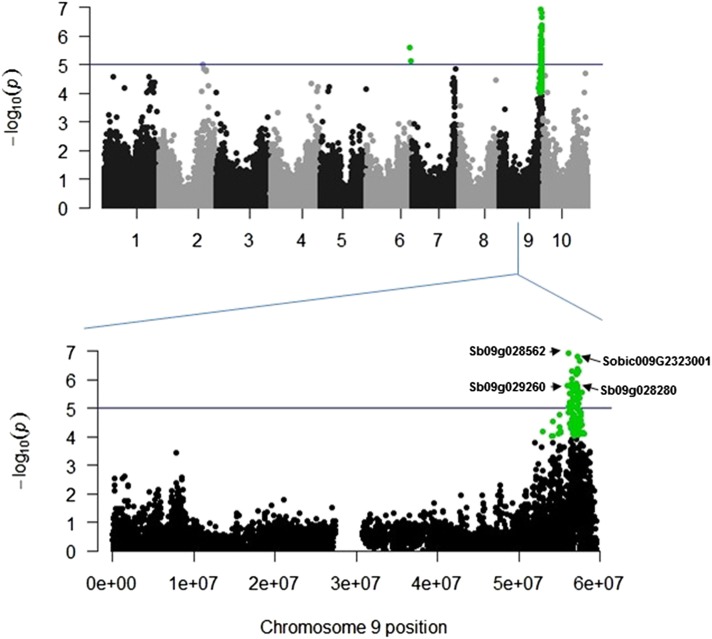
Association result for stalk rot resistance in a panel of 300 genotypes based on 79,132 SNPs. (A) Physical map locations of the SNPs (x-axis), the –log base 10 *P* values from a mixed linear model (y-axis), and the Bonferroni-adjusted significance threshold based on effective number of independent tests (blue horizontal line). (B) Regional plots showing association mapping results for significantly associated SNPs located on chromosome 9. Plots are based on pooled significant SNPs for all stalk rot traits.

The genetic basis of variation for stalk rot resistance in sorghum was examined using a unified mixed linear model that controls for population structure and familial relatedness. Three SNPs were significant for at least one trait at a genome-wide Bonferroni-corrected threshold that was estimated based on an effective number of independent tests (Figure 3, Figure S1, Figure S2, Figure S3, Figure S4, Figure S5, Figure S6, Figure S7, Figure S8, Figure S9, Figure S10, Figure S11, and Table S2). Given the complex nature of the inheritance of stalk rot resistance traits in sorghum, and also given that most of the significant SNPs in the unified mixed model were located within a 2-Mb region on chromosome 9 (Figure 3 and Table S2), we used the MLMM of Segura *et al.* (2012) on a chromosome-wide scale to further dissect the complex association signals that we observed for stalk rot resistance (Table 3, Figure 3, Figure S1, Figure S2, Figure S3, Figure S4, Figure S5, Figure S6, Figure S7, Figure S8, Figure S9, Figure S10, and Figure S11). Based on the MLMM, we identified 14 major effect loci associated with stalk rot resistance from the combined environment as well as individual location analyses. In the combined analysis, the optimal model detected two significant SNPs. One of the SNPs (S9_57816733) was significantly associated with MLL and TLL for *F. thapsinum* (Figure S1b and Figure S3b). Another SNP (S9_57222599) from the optimal model was also significant for both MLL and TLL for *M. phaseolina* (Figure S2b and Figure S4b). These SNPs had the strongest significant association signals in the unified mixed model (Table S2). The two SNPs individually accounted for 11% to 16% of the total phenotypic variation explained by the traits and the two SNPs are in linkage equilibrium (r^2^ = 0.45) with each other (Figure S12).

**Table 3:** Chromosome locations, allele effect estimates, genes containing or adjacent to SNP, and other summary statistics for SNPs significantly associated to stalk rot resistance in the combined, and individual environment analysis from the multi-locus mixed model

Gene Containing or Adjacent to SNP	SNP Physical Position (bp)	*P*	Disease Traits	Allele	N*^a^*	Allele Effect*^b^*	*R*^2^*^c^*	Annotated Gene Function
**Combined analysis**								
Sb09g029260	Chr 9: 57816733	8.87E-07	MLL (*FT*)	A	174	1.14	0.12	Chalcone synthase
				G	61	—		
Sb09g028280.1	Chr 9: 57222599	7.01E-09	MLL (*MP*)	C	169	1.20	0.16	ROP GTPase proteins
				G	57	—		
Sb09g029260	Chr 9: 57816733	6.98E-07	TLL (*FT*)	A	174	1.50	0.11	Chalcone synthase
				G	61	—		
Sb09g028280.1	Chr 9: 57222599	8.60E-09	TLL (*MP*)	C	169	1.30	0.16	ROP GTPase proteins
								
**Manhattan combined**								
Sb09g028562	Chr 9: 57272115	1.18E-07	MLL (*MP*)	A	58	0.84	0.13	Transcription factor
				G	186	—		
Sb07g021660	Chr 7:55702883	1.42E-07	RMLL (*FT*)	T	154	0.99		Protein kinase
				G	62	—		
Sb02g029630	Chr 2:64623580	2.74E-07	RMLL (*FT*)	G	122	0.99	0.24	Auxin-induced protein
				A	91	—		
Sb09g028562	Chr 9:57272115	3.68E-07	TLL (*FT*)	A	58	0.87	0.13	Transcription factor
				G	186	—		
Sb09g028562	Chr 9:57272115	1.23E-07	TLL (*MP*)	A	58	0.83	0.14	Transcription factor
				G	186	—		
Sb02g025370	Chr 2:60129082	3.84E-10	RMLL (*MP*)	C	133	0.99		PPR protein
				T	84	—		
Sb07g023700	Chr 7:58532122	8.28E-09	RMLL (*MP*)	G	203	0.99		Unknown function
				A	30	—		
Sobic.009G232300.1	Chr 9:57231207	5.70E-08	RMLL (*MP*)	T	213	0.99		Transcription factor
				C	13	—		
Sb02g014480	Chr 2:27851064	5.13E-06	RMLL (*MP*)	A	153	1.00	0.33	Protein binding
				G	76			
**Ottawa**								
Sb03g008300	Chr 3:8738404	1.63E-08	RTLL (MP)	A	200	1.00		Zinc finger protein
				G	27	—		
Sb09g027770	Chr 9:56646280	1.06E-07	RTLL (MP)	A	84	—		Unknown function
				T	149	0.99		
Sb04g036090	Chr 4:65815798	1.20E-06	RTLL (MP)	T	211	0.99		Signal transduction
				C	17	—		
Sb08g020320	Chr 8:51279094	5.23E-06	RTLL (MP)	C	129	0.99	0.34	Protein binding
				T	79	—		
sobic.008G098200.1	Chr 8:39230116	1.18E-06	TLL (FT)	G	203	1.21	0.13	Unknown function
				A	21	—		

TLL, total lesion length; MLL, major lesion length; RTLL, relative total lesion length; RMLL, relative major lesion length. MP, *Macrophomina phaseolina*; FT, *Fusarium thapsinum*.

*^a^* N is the total number of lines with the specific SNP genotype. *^b^*Allele effects back-transformed to the original scale in millimeters. *^c^R*^2^, proportion of total line mean variance explained by SNP as computed by GAPIT.

Because of the significant G × E observed in the overall combined analysis, we also conducted an independent association study for the Manhattan (two seasons combined) and Ottawa locations to capture location-specific loci associated with pathogen response. Analysis of combined data from the Manhattan location identified one additional significant SNP (S9_57272115) associated with TLL in both *M. phaseolina* and *F. thapsinum* and with MLL in *M. phaseolina* (Figure S5b, Figure S8b, and Figure S9b). This SNP accounted for approximately 14% of the phenotypic variation observed for each of these traits (Table 3). This SNP also had the strongest association signal for the three traits in the unified mixed model (Table S2). Two additional SNPs associated with MLL in *F. thapsinum* (S2_64623580 and S7_55702883) were identified with the forward-backward stepwise approach of MLMM (Figure S6b). The two SNPs accounted for 24% of the total phenotypic variation for the trait (Table 3). Using the same approach, four other SNPs (S2_27851064, S2_60129082, S7_58532122, and S9_57231207) were found to be associated with relative MLL in *M. phaseolina* (Figure S7b). The four SNPs accounted for 33% of the total phenotypic variation for RMLL (Table 3). For the Ottawa location, four additional SNPs (S3_8738404, S4_65815798, S8_51279094, and S9_56646280) were found to be associated with relative TLL in *M. phaseolina* (Figure S10b), which together accounted for 34% of the total phenotypic variation observed for the trait. Similarly, one additional SNP (S8_39230116) was associated with TLL in *M. phaseoina*, which also accounted for 13% of the total phenotypic variation (Table 3; Figure S11b). Clearly, stalk rot resistance is subject to genotype × environment interactions, which might be related to the severity of the stress. Such effects will be reflected in the QTL × environment interaction leading to SNPs being significant at one environment but not at the other.

### Linkage disequilibrium

Classic LD parameters (D′ and r^2^) were used to test whether the significant SNPs were in strong LD with each other (Figure S12). The presence of very strong LD would raise concerns about a high rate of false positives present in our results, whereas complete absence of LD between the significant SNPs would provide evidence of complete independence between QTL. We observed significant LD only between SNPs S9_57222599 and S9_57272115 (r^2^= 0.52), both located on chromosome 9 at 57222599 and 57272115 Mb, respectively. For the rest of the SNPs, we did not observe signs of strong inter-QTL LD resulting from population substructure and admixture within the panel even for closely linked significant SNPs observed in the combined analysis such as S9_57222599 and S9_57816733 both mapping on chromosome 9 (r^2^ = 0.20). With an average r^2^ value of 0.06 for all 91 possible combinations between the 14 significantly associated SNPs, most observed r^2^ values were <0.30. Only four, which were among the six possible combinations between SNPs S9_57222599, S9_57231207, S9_57272115, and S9_57816733, were greater than 0.3 and none exceeded 0.5 (Figure S12).

### Allele distribution at significant SNPs

Allele frequencies at the 14 SNPs significantly associated with stalk rot resistance across the four major sorghum subpopulations were estimated (Table 4). In the combined analysis, the allele that reduced disease severity at SNP locus S9_57816733 on chromosome 9 was over-represented in the caudatum and durra subpopulations compared to the guinea and kafir subpopulations (*P* = 3.01 × 10^−4^). The second SNP, S9_57222599, on the same chromosome is represented more or less equally across all subpopulations.

**Table 4:** Allele frequencies of significantly associated SNPs for overall combined, Manhattan combined, and Ottawa environments in the four major subpopulations

Chromosome	SNP Physical Position (bp)	Resistant Allele	Allele Frequency (%)				*P*	N			
			CAD	DUR	GUI	KAF		CAD	DUR	GUI	KAF
**Overall combined**											
**9**	57816733	G	41.2	27.8	9.5	0.0	3.01E-04	51	36	21	24
**9**	57222599	G	35.3	16.7	9.5	20.8	1.29E-01	51	36	21	24
**Manhattan combined**											
**9**	57272115	G	60.8	97.2	66.7	70.8	8.12E-04	51	36	21	24
**7**	55702883	G	72.6	8.3	0.0	0.0	2.20E-16	51	36	21	24
**2**	64623580	A	37.3	66.7	9.5	8.3	1.41E-07	51	36	21	24
**2**	60129082	T	11.7	36.1	71.4	58.3	4.00E-06	51	36	21	24
**7**	58532122	A	5.9	22.2	4.8	4.2	4.22E-02	51	36	21	24
**9**	57231207	C	13.7	0.0	9.5	0.0	1.33E-02	51	36	21	24
**2**	27851064	G	2.0	69.4	85.7	12.5	2.20E-16	51	36	21	24
**Ottawa**											
**3**	8738404	G	9.8	2.8	0.0	8.3	2.55E-04	46	34	21	24
**9**	56646280	A	39.2	19.9	28.5	37.5	9.99E-03	44	34	21	24
**4**	65815798	C	0.0	27.8	0.0	0.0	1.53E-06	50	33	21	24
**8**	51279094	T	27.4	69.4	52.4	4.0	5.41E-08	44	29	21	24
**8**	39230116	A	0.0	30.6	0.0	0.0	5.41E-08	47	36	21	24

SNP, single nucleotide polymorphism; N, total number of lines within each subpopulation; CAD, caudatum; DUR, durra; GUI, guinea; KAF, kafir.

The frequency of allele at SNP S9_57272115 associated with reduced disease severity in the combined analysis for Manhattan location was high in all subpopulations. The resistance allele in the second significant SNP (S9_57231207) was represented only in the caudatum and guinea subpopulations. On chromosome 7, the allele associated with low disease severity at SNP S7_55702883 was significantly over-represented among the caudatum subpopulation (*P* = 2.02 × 10^−16^) and the one at SNP S7_58532122 in the durra subpopulation (*P* = 0.0013). Also, the frequency of the allele associated with resistance at the SNP located on chromosome 2 (S2_64623580) from the Manhattan combined data were again largely represented in the cadutaum and durra subpopulations (*P* = 1.41 × 10^−7^). For the other SNPs on chromosome 2 (S2_60129082), the allele reducing disease severity was significantly (*P* = 4 ×10^−6^) over-represented in the guinea and kafir subpopulations compared to the caudatum and durra. Another allele at SNP S2_27851064 on the same chromosome was significantly (*P* = 2 × 10^−16^) over-represented in the durra and guinea subpopulations and was much less in caudatum and kafir groups.

In the Ottawa analysis, the allele involved in reduced disease severity on SNP S3_8738404 was only present in the caudatum, durra, and kafir subpopulations, whereas other alleles at SNP S8_39230116 and S4_65815798 were only present or significantly (*P* = 5.14 × 10^−8^) over-represented in the durra subpopulation. Also on chromosome 8, the allele at SNP S8_51279094 reducing disease severity was significantly (*P* = 5.14 × 10^−8^) over-represented in the durra and guinea, whereas the SNP on chromosome 9 (S9_57231207) was present and significantly (*P* = 14 × 10^−8^) over-represented only in the caudatum and guinea subpopulations (Table 4).

### Candidate genes co-localized with associated SNPs

Two genes identified as part of SNPs S9_57272115 and S9_57816733 (Table 3) in the combined analysis were shown to have predicted gene functions related to immune response pathways, including a ring finger domain containing protein, tyrosine kinase protein, chalcone, and stilbene synthases (Dao *et al.* 2011). Genes contained within significant SNPs associated with reduced disease severity in combined data from the Manhattan location also have predicted functions such as AP2 domain containing protein, a homobox domain containing protein, protein kinase domain, auxin-induced protein, UDP-glucuronosyltransferase gene, and pentatricopeptide repeat-containing protein. Similarly, genes that are part of or adjacent to SNPs S3_8738404 associated with stalk rot resistance were revealed, from the analysis of the Ottawa data, to be located in an intronic region of hAT family dimerization domain with similarity to the zinc finger protein. Other genes associated with SNP S4_65815798 and S8_51279094 were located in the region of an acetylglucosaminyltransferase gene family and one coding for sterol regulatory element-binding (SERP) protein domain.

## Discussion

### Phenotypic variation and correlations

The present study utilizes genome-wide association mapping to locate and map genes or genomic regions associated with resistance to stalk rot diseases caused by two primary causal organisms, *M. phaseolina* and *F. thapsinum*. The germplasm used in this study represents the global sorghum diversity and is an excellent resource for GWAS. The quality of the phenotypic data has significant bearing on the accuracy of GWAS (Rafalski 2010) and on the applicability of the markers thus identified. A common concern in screening of such genotypes for stalk rot resistance is that differences in maturity among the test population may confound disease response as genotypes of various maturity lengths may be exposed to array of environmental variables of different intensity. This may significantly influence genotypic responses to stalk rot diseases because host plant responses are highly dependent on the extent of environmental stress and the growth stages at which the stresses are initiated. To account for this, days to flowering was considered as a fixed linear covariate during the analysis. Through the use of relative lesion length measures, similar effort was made to circumvent the potential bias that may arise as a result of differences in plant height when disease severity is rated as lesion length.

Taking these together, the present study showed marked variability for response to both *M. phaseolina*–induced and *F. thapsinum*–induced stalk rot diseases. Repeatability on a genotype mean basis ranged from 0.62 to 0.97. Given the complex nature of the disease and difficulty related to administering the treatments and scoring the data, the level of repeatability observed was high for all traits, indicating that biologically meaningful QTL are responsible for much of the phenotypic variation reflected in the population. Thus, the use of markers associated with the QTL responsible for resistance to these diseases should increase efficiency of selection and therefore contribute to accelerating breeding efforts to develop germplasm enhanced for resistance to these diseases. One of the major constraints in breeding for stalk rot resistance besides the tedious field inoculation and disease scoring is the likely confounding effect of disease scoring (*i.e.*, lesion length) with plant height. Although the number of nodes crossed was often used to account for this, genotypes with variable internode length always appear different in their reaction to stalk rot, even if same number of nodes were crossed. Here, we introduced the parameter “relative lesion length,” which is the ratio of length of infection (mm) to plant height (cm) to circumvent this problem. However, the repeatability of these derived traits (RTLL and RMLL) for both *M. phaseolina* and *F. thapsinum* on a genotype mean basis was lower than the corresponding disease severity measures (TLL and MLL) for both pathogens. The repeatability estimate was obtained by modeling each genotype as a random sample from the reference population, modeled by a genotypic variance–covariance structure equal to the genotypic variance multiplied by an identity matrix. Thus, the possible reasons for low repeatability for the derived traits may be due to the lower variation for these traits in the population have been accounted for by plant height. Moreover, environmental stresses that increase disease severity such as drought and high-temperature stress tend to reduce plant height, resulting in inconsistent relative lesion length across environments and reducing repeatability.

Although the two pathogens associated with stalk rot disease are biologically distinct, the response of genotypes to infection by both pathogens was strongly correlated (Table 2). This may be the result of similarity in both environmental and biological events that leads to disease development in both pathogens. Although charcoal rot is considered more aggressive than *Fusarium* stalk rot, both diseases are triggered by high temperature and prolonged drought conditions during crop maturation (Zummo 1984). Moreover, genetic mechanisms of the host plant controlling these traits may be similar for both pathogen groups. Previous reports have suggested that some genotypes with charcoal rot resistance could also be resistant to *Fusarium* stalk rot (Tesso *et al.* 2010; Bramel-Cox *et al.* 1988). Both TLL and MLL in *M. phaseolina* and *F. thapsinum* were moderately correlated with plant height, suggesting the possible confounding effect of internode length. But there was no correlation between the derived traits (RTLL and RMLL) and plant height (Table 2), indicating that the derived traits may be beneficial to account for the potential bias that differences in plant height may introduce while scoring disease severity.

### Association analysis

Association mapping of the SNP markers on the disease phenotype data pooled across all environments identified two SNPs significantly associated with low TLL and MLL in *M. phaseolina* (S9_5816733, SNP1) and *F. thapsinum* (S9_57222599, SNP2), both located on chromosome 9. But none of these SNPs was significant when data were analyzed separately for each individual location. However, a different set of SNPs located on the same chromosome, S9_57272115 (SNP3) and S9_57231207 (SNP4) for Manhattan data and S9_56646280 (SNP5) for Ottawa data, became significant for both TLL and MLL for both pathogen species. Although the two SNPs that were significant in the combined analysis were not significant in individual location data, these five SNPs were within a 1-Mb region on chromosome 9. There appeared to be a relatively high LD between SNP1 and SNP2 (r^2^ = 0.52) and between SNP2 and SNP3 (r^2^ = 0.45). Thus, it is possible that many of these SNPs are associated with the same underlying causal variation. However, SNP4 and SNP5 are in low LD with the other SNPs, suggesting that they are distinct from the causal polymorphisms with which the other SNPs are associated.

Analysis of these SNPs in relation to previous published results indicates that they co-localize with the previously described plant height locus dw1/SbHt9.1 (57272115 Mb) (Brown *et al.* 2008). This locus is 29 kb away from a GA2 oxidase, a catabolic enzyme in the gibberellin pathway that is proposed as the gene underlying plant height QTL SbHt9.1/Dw1 (Wang *et al.* 2012b). Although there is no information about the relationship between stalk rot resistance and GA3, such an association may imply that lesion length–based stalk rot reaction may be associated with plant height. We were concerned with such a possibility from the beginning of the study and introduced a parameter that accounts for differences in plant height. These parameters, RTLL and RMLL, which had no correlation with plant height (as mentioned above), also mapped to this region, indicating that these SNPs were associated with genetic causes directly associated with stalk rot response. Furthermore, SNPs identified as significantly associated with stalk rot resistance are located within regions where genes with predicted functions related to the immune response pathway are located.

The effect of environment on stalk rot disease incidence and severity has been reported to be very significant (Tesso *et al.* 2010), and the different QTL identified across environments agree with these previous reports. The fact that all of the significant QTL occur in the same region on chromosome 9 indicates that although disease severity across environments can be different, genotypic reaction may be relatively consistent across locations. Previous studies conducted on a smaller set of genotypes across environments have shown that while ratings can vary, resistant genotypes tend to express resistance reaction across environments (Tesso *et al.* 2005). However, due to the difficulty with inoculation and disease scorings, only a limited number of environments were sampled in the present study. Future work should consider suitably augmenting the optimal level of genetic diversity and number of test environments to shed light on the role that the QTL × environment interaction could play in affecting disease severity.

Although the SNPs explained a relatively large portion of the total variation on genotype means, each SNP had a relatively small additive effect on stalk rot resistance, with a range of 0.84 to 1.50. In every case, an increase in disease resistance was associated with a rare allele at each locus. Moreover, the frequency of alleles associated with enhanced resistance to stalk rot was higher in the caudatum subpopulation relative to other groups for the combined analysis, whereas they were over-represented in the durra subpopulation for the individual location analysis. Durra sorghums predominantly occur in the warm semi-arid climates of the Horn of Africa, Arabian Peninsula, and India (Morris *et al.* 2013). Characterized by a very compact inflorescence, they are the primary sources of drought tolerance, with many drought-associated QTL mapped in diverse genetic backgrounds of durra derivative. Because drought tolerance has been repeatedly demonstrated to be associated with stalk rot resistance (Crasta *et al.* 1999; Rosenow *et al.* 1977; Rosenow *et al.* 1983), the current finding agrees with these previous reports that show that durra sorghums harboring a higher frequency of alleles associated with stalk rot resistance.

### Candidate genes

Using the sorghum reference genome sequence, we attempted to identify genes that either were included or were near SNPs significantly associated with stalk rot resistance. One of the two SNPs identified on chromosome 9 (S9_57816733) in the combined data were linked to the Sb09g029260 gene that belongs to the chalcone and stilbene synthase protein family. Chalcone synthase is a key enzyme in the flavonoid/isoflavonoid biosynthesis pathway. Besides being part of the plant developmental program, chalcone synthase gene expression is induced in plants under stress conditions such as UV light and bacterial or fungal infection. Chalcone synthase expression causes accumulation of flavonoid and isoflavonoid phytoalexins and is involved in the salicylic acid defense pathway (Dao *et al.* 2011). The second gene (Sb09g028280.1) linked to SNP S9_57222599 belongs to the family of ROP GTPase proteins, an important signaling molecule that regulates hormone responses and disease resistance in plants (Tao *et al.* 2002; Ono *et al.* 2001; Kawasaki *et al.* 1999).

The SNP identified on chromosome 9 from the Manhattan combined data was associated with another gene (Sobic.009G233100.1) that belongs to the AP2 transcription factor family found only in plants. This gene has been reported to code for proteins involved in regulation of disease resistance pathways (Gutterson and Reuber 2004). Similarly, another gene (Sb07g021660) linked to SNP on chromosome 7 encodes for protein kinases also known to be involved in disease response. On chromosome 2, (Sb02g029630) codes for a protein that is similar to auxin-induced proteins. Also on chromosome 2, (Sb02g025370) has been reported to code for pentatricopeptide repeat (PPR)-containing proteins. PPR genes show features common to disease resistance genes (Foxe and Wright 2009). In Arabidopsis, PPR proteins have been shown to play a defense role against neurotropic fungi and abiotic stress tolerance (Laluk *et al.* 2011). Another gene (Sb09g028320.1) linked to a significant SNP on chromosome 9 is from the UDP-glucuronosyltransferase family of proteins. Altering glucosinolate profiles in plants have been shown to modulate disease resistance (Brader *et al.* 2006).

Significant SNPs identified on chromosome 8 (S8_39230116) and chromosome 9 (S9_5664280) from the Ottawa data were located within genes of unknown function, (sobic.008G098200.1) and 9 (Sb09g027770), respectively. The other SNP on chromosome 3 (S3_8738404) is located downstream of gene (Sb03g008300) and codes for a BED zinc finger protein domain that is a major component of R proteins (Aravind 2000; Tuskan *et al.* 2006). The SNP on chromosome 4 is located upstream of a gene (Sb04g036090). It codes for an acetylglucosaminyltransferase protein, a major component of signal transduction pathways leading to systemic acquired resistance in plants (Klessig and Malamy 1994). Infection of plants by necrotizing pathogens may lead to the induction of a complex set of defense responses, resulting in restriction of pathogen growth and spreading. Infected leaves develop a hypersensitive response (HR), a rapid, localized cell death occurring at the sites of pathogen entry (Heath 2000). Also associated with this gene are the accumulation of salicylic acid and several classes of pathogenesis-related (PR) proteins that exhibit antimicrobial activity (Ward *et al.* 1991). The SNP on chromosome 8 is located downstream of gene Sb08g020320 coding for a sterol regulatory element–binding protein.

In conclusion, progress in the understanding of the genetic basis of stalk rot resistance in sorghum is complicated by multiple factors. The difficulty of reproducing consistent field environments and the low-throughput phenotyping protocols currently used are among the limitations to breeding for stalk rot resistance. The current study is the first effort to apply GWAS to understand the genetic mechanisms underlying stalk rot disease resistance in sorghum. We generated 70,000 SNP marker data and robust charcoal rot and *Fusarium* stalk rot phenotype data in the global sorghum association panel and integrated those to discover and map loci associated with resistance to both diseases. We identified 14 loci, some of which were associated with a set of candidate genes, that appear to be involved in a set of functions controlling the plant defense response. This research serves as a basis for the identification of stalk rot resistance or defense-associated genes that advances our understanding of the complicated molecular mechanisms that regulate the plant response to *M. phaseolina* and *F. thapsinum*.

## Supplementary Material

Supporting Information
