# Supplementary material for: Genome-Wide Association Study on Resistance to Stalk Rot Diseases in Grain Sorghum
Source: G3 (Bethesda). 2015 Apr 16;5(6):1165–75. doi: 10.1534/g3.114.016394 (PMC4478546; doi:10.1534/g3.114.016394)
Supplement: Supporting Information [file supp_5_6_1165__index.html]

Genome-Wide Association Study on Resistance to Stalk Rot Diseases in Grain Sorghum — Supporting Information 

# Genome-Wide Association Study on Resistance to Stalk Rot Diseases in Grain Sorghum

## Supporting Information for Adeyanju *et al.*, 2015

**Files in this Data Supplement:**

- Supporting Information - Tables S1-S4, Figures S1-S12, and Files S1-S2 (PDF, 1 MB)
- Table S1 - Genotypic covariance/ variance /correlation matrix for stalk rot traits from the combined analysis of variance for three environments. (PDF, 165 KB)
- Table S2 - Chromosome locations, and other summary statistics for SNPs significantly associated with stalk rot resistance in the overall combined, Manhattan combined and Ottawa performed using the unified mixed model. (PDF, 115 KB)
- Table S3 - List of sorghum genotypes included in the population structure analysis (based on 25,000 SNPs) showing the subpopulation assignment and probability values of membership. (PDF, 122 KB)
- Table S4 - Chromosome locations, and other summary statistics for SNPs significantly associated with stalk rot resistance in the combined, Manhattan combined and Ottawa analysis based on the unified mixed model. (PDF, 115 KB)
- Figure S1 - Plots of SNPs associated with major lesion length (MLL) for *Fusarium thapsinum* based on the combined analysis. (PDF, 188 KB)
- Figure S2 - Plots of SNPs associated with major lesion length (MLL) for *Macrophomina phaseolina* based on the combined analysis. (PDF, 184 KB)
- Figure S3 - Plots of SNPs associated with major lesion length (TLL) for *Fusarium thapsinum* based on the combined analysis. (PDF, 195 KB)
- Figure S4 - Plots of SNPs associated with total lesion length (TLL) for *Macrophomina phaseolina* based on the combined analysis. (PDF, 198 KB)
- Figure S5 - Plots of SNPs associated with total lesion length (TLL) for *Macrphomina phaseolina* based on across year combined data for Manhattan locations. (PDF, 211 KB)
- Figure S6 - Plots of SNPs associated with relative major lesion length (RMLL) for *Fusarium thapsinum* based on across year combined data for Manhattan locations. (PDF, 212 KB)
- Figure S7 - Plots of SNPs associated with relative major lesion length (RMLL) for *Macrphomina phaseolina* based on across year combined data for Manhattan locations. (PDF, 194 KB)
- Figure S8 - Plot of SNP S9\_57272115 associated with total lesion length (TLL) for *Fusarium thapsinum* based on across year combined data for Manhattan locations. (PDF, 193 KB)
- Figure S9 - Plot of SNP S9\_57272115 associated with total lesion length (TLL) in *Macrphomina phaseolina* based on across year combined data for Manhattan locations. (PDF, 192 KB)
- Figure S10 - Plots of SNPs associated with relative total lesion length (RTLL) caused by inoculation with *Macrphomina phaseolina* based on data for Ottawa locations. (PDF, 199 KB)
- Figure S11 - Plots of SNPs associated with relative total lesion length (RTLL) caused by inoculation with *Fusarium thapsinum* based on data for Ottawa locations. (PDF, 192 KB)
- Figure S12 - Linkage Disequilibrium (LD) among significant SNP markers. (PDF, 128 KB)
- File S1 - Raw phenotypic data from combined environment, Manhattan combined environments, and and Ottawa environment. Formatted for analysis in ASReml software. Columns in the data file from left to right are as follows: environment (Env, a unique combination of location and year), Year (1=2011, 2=2012), Rep, line, number of plants scored within each plot for the *Macrophomina* pathogen(NM), logTLM = natural log transformation of the total Lesion length *Macrophomina*, logRMLM= natural log transformation of the relative major Lesion length *Macrophomina*, logMLM = natural log transformation of the major Lesion length *Macrophomina*, NNCM= Number of nodes crossed by the *Macrophomina* pathogen, number of plants scored within each plot for the *Fusarium* pathogen(NF), logTLF = natural log transformation for the total Lesion length *Fusarium*, logRMLF= natural log transformation of the relative major Lesion length *Fusarium* logMLF= natural log transformation of the major Lesion length *Fusarium*, NNCF= Number of nodes crossed by the *Fusarium* pathogen, PH= Plant height, DFF=Days to 50% Flowering. (.csv, 409 KB)
- File S2 - Least square means for 300 inbred lines estimated within each environment (OTTAWA) and across environments. (.csv, 73 KB)
