# Supplementary material for: Genome-Wide Association Study on Resistance to Stalk Rot Diseases in Grain Sorghum
Source: G3 (Bethesda). 2015 Apr 16;5(6):1165–75. doi: 10.1534/g3.114.016394 (PMC4478546; doi:10.1534/g3.114.016394)
Supplement: Supporting Information [file supp_g3.114.016394_TableS1.pdf]

Supplementary Tables

Table S1 Genotypic covariance/ variance /correlation matrix for stalk rot traits from the combined analysis of variance for three environments. The diagonal (bold) is an estimate of genetic variance ($\hat{\sigma}G^2$) plus the genotype by environment interaction ($\hat{\sigma}GE^2$) within each environment. Estimates of covariance between pairs of environments are shown below the diagonal, and genetic correlations between genotypes in each pair of environments are shown above the diagonal.

	Fusarium thapsinum			Macrophomina phaseolina		
	Manhattan			Manhattan		
	2011	2012	Ottawa 2012	2011	2012	Ottawa 2012
Major lesion length						
Manhattan 2011	**0.14**	0.83	0.66	**0.17**	0.89	0.51
Manhattan 2012	0.11	**0.12**	0.72	0.14	**0.15**	0.66
Ottawa 2012	0.09	0.09	**0.12**	0.10	0.13	**0.24**
Total lesion length						
Manhattan 2011	**0.14**	0.75	0.48	**0.18**	0.89	0.57
Manhattan 2012	0.10	**0.13**	0.66	0.16	**0.17**	0.68
Ottawa 2012	0.07	0.09	**0.14**	0.13	0.15	**0.29**
Relative total lesion length						
Manhattan 2011	**0.11**	0.70	0.36	**0.12**	0.84	0.44
Manhattan 2012	0.08	**0.13**	0.52	0.10	**0.12**	0.47
Ottawa 2012	0.04	0.06	**0.11**	0.07	0.08	**0.22**
Relative major lesion length						
Manhattan 2011	**0.10**	0.77	0.56	**0.11**	0.85	0.35
Manhattan 2012	0.08	**0.11**	0.56	0.09	**0.10**	0.44
Ottawa 2012	0.05	0.05	**0.09**	0.05	0.06	**0.18**

Plant height				***Days to flowering***		
Manhattan 2011	**587.70**	0.79	0.88	**0.33**	0.76	0.82
Manhattan 2012	613.80	**1029.00**	0.99	0.20	**0.21**	0.76
Ottawa 2012	318.00	473.10	**223.30**	0.30	0.22	**0.39**
