# Supplementary material for: Genome-Wide Association Study on Resistance to Stalk Rot Diseases in Grain Sorghum
Source: G3 (Bethesda). 2015 Apr 16;5(6):1165–75. doi: 10.1534/g3.114.016394 (PMC4478546; doi:10.1534/g3.114.016394)
Supplement: Supporting Information [file supp_g3.114.016394_TableS2.pdf]

**Table S2 Chromosome locations, and other summary statistics for SNPs significantly associated with stalk rot resistance in the overall combined, Manhattan combined and Ottawa performed using the unified mixed model.**

| Chromosome | SNP Physical Position, bp | P-Value | MAF[a] | Disease traits[b] |
|---|---|---|---|---|
| Combined analysis | | | | |
| 9 | 57816733 | 2.87E-06 | 0.28 | MLL (*FT*) |
| 9 | 57272115 | 4.18E-06 | 0.25 | MLL (*FT*) |
| 6 | 60030948 | 5.65E-06 | 0.10 | MLL (*FT*) |
| 9 | 57222599 | 1.48E-07 | 0.28 | MLL (*MP*) |
| 9 | 57476134 | 2.28E-07 | 0.30 | MLL (*MP*) |
| 9 | 57272296 | 5.58E-07 | 0.27 | MLL (*MP*) |
| 9 | 56152890 | 6.56E-07 | 0.34 | MLL (*MP*) |
| 9 | 57169768 | 7.50E-07 | 0.23 | MLL (*MP*) |
| 9 | 57272115 | 7.72E-07 | 0.25 | MLL (*MP*) |
| 3 | 60176979 | 4.97E-05 | 0.23 | RMLL (*FT*) |
| 2 | 64623580 | 9.16E-05 | 0.44 | RMLL (*FT*) |
| 7 | 58280357 | 1.43E-05 | 0.35 | RMLL (*MP*) |
| 7 | 56256841 | 1.95E-05 | 0.39 | RMLL (*MP*) |
| 3 | 60176979 | 7.62E-05 | 0.23 | RTLL (*FT*) |
| 7 | 56152038 | 1.35E-05 | 0.47 | RTLL (*MP*) |
| 9 | 56152890 | 1.87E-05 | 0.34 | RTLL (*MP*) |
| 9 | 57272115 | 1.72E-06 | 0.25 | TLL (*FT*) |
| 9 | 57222599 | 2.07E-06 | 0.28 | TLL (*FT*) |
| 9 | 57816733 | 2.66E-06 | 0.28 | TLL (*FT*) |
| 9 | 57222599 | 8.93E-08 | 0.28 | TLL (*MP*) |
| 9 | 56152890 | 1.17E-07 | 0.34 | TLL (*MP*) |
| 9 | 57476134 | 2.25E-07 | 0.30 | TLL (*MP*) |
| 9 | 56508161 | 4.94E-07 | 0.41 | TLL (*MP*) |
| 9 | 57236778 | 5.82E-07 | 0.29 | TLL (*MP*) |
| 9 | 57272296 | 6.91E-07 | 0.27 | TLL (*MP*) |
| 9 | 57272115 | 7.47E-07 | 0.25 | TLL (*MP*) |
| 9 | 56562984 | 9.71E-07 | 0.34 | TLL (*MP*) |
| Manhattan combined analysis | | | | |
| 9 | 57272115 | 2.93E-06 | 0.25 | MLL (*FT*) |
| 9 | 57816733 | 3.36E-06 | 0.28 | MLL (*FT*) |
| 9 | 57383556 | 6.39E-06 | 0.30 | MLL (*FT*) |
| 9 | 57236791 | 6.70E-06 | 0.30 | MLL (*FT*) |
| 9 | 57272115 | 4.04E-07 | 0.25 | MLL (*MP*) |
| 9 | 57272296 | 6.16E-07 | 0.27 | MLL (*MP*) |
| 9 | 57476134 | 6.53E-07 | 0.30 | MLL (*MP*) |
| 9 | 57169768 | 6.69E-07 | 0.23 | MLL (*MP*) |
| 9 | 57383556 | 9.89E-07 | 0.30 | MLL (*MP*) |

| Chromosome | SNP Physical Position, bp | P-Value | MAF[a] | Disease traits[b] |
|---|---|---|---|---|
| 2 | 60129082 | 9.67E-06 | 0.40 | RMLL (*MP*) |
| 6 | 59739236 | 1.89E-05 | 0.46 | RTLL (*FT*) |
| 2 | 64623580 | 1.61E-05 | 0.44 | RMLL (*FT*) |
| 7 | 59890439 | 1.07E-05 | 0.32 | RTLL (*MP*) |
| 9 | 57272115 | 2.48E-06 | 0.25 | TLL (*FT*) |
| 9 | 57816733 | 5.56E-06 | 0.28 | TLL (*FT*) |
| 9 | 57236791 | 6.72E-06 | 0.30 | TLL (*FT*) |
| 9 | 56152890 | 2.82E-07 | 0.34 | TLL (*MP*) |
| 9 | 57272115 | 4.34E-07 | 0.25 | TLL (*MP*) |
| 9 | 57383556 | 4.99E-07 | 0.30 | TLL (*MP*) |
| 9 | 57222599 | 5.93E-07 | 0.28 | TLL (*MP*) |
| 9 | 57476134 | 9.28E-07 | 0.30 | TLL (*MP*) |
| Ottawa analysis | | | | |
| 1 | 11814753 | 1.70E-05 | 0.12 | MLL (*FT*) |
| 4 | 55646941 | 1.78E-05 | 0.31 | MLL (*FT*) |
| 2 | 69967056 | 3.73E-05 | 0.17 | MLL (*MP*) |
| 1 | 42155829 | 5.19E-05 | 0.10 | MLL (*MP*) |
| 3 | 60195553 | 4.48E-05 | 0.31 | RMLL (*FT*) |
| 4 | 55646941 | 6.59E-05 | 0.31 | RMLL (*FT*) |
| 1 | 66836006 | 1.20E-05 | 0.08 | RMLL (*MP*) |
| 8 | 51279094 | 2.55E-05 | 0.40 | RMLL (*MP*) |
| 6 | 54221186 | 6.27E-05 | 0.17 | RTLL (*FT*) |
| 10 | 3137796 | 8.26E-05 | 0.17 | RTLL (*FT*) |
| 1 | 61119284 | 2.68E-05 | 0.32 | RTLL (*MP*) |
| 8 | 51279094 | 3.48E-05 | 0.40 | RTLL (*MP*) |
| 10 | 3137796 | 2.38E-05 | 0.17 | RTLL (*FT*) |
| 9 | 56802949 | 2.61E-05 | 0.18 | RTLL (*FT*) |
| 1 | 27040589 | 6.39E-05 | 0.32 | TTL (*MP*) |
| 1 | 61119285 | 6.57E-05 | 0.32 | TTL (*MP*) |

[a]MAF, minor allele frequency.

[b]Disease traits: TLL and MLL – total lesion length and major lesion length, respectively; RTLL and RMLL – relative total lesion length and relative major lesion length, respectively; *MP* and *FT* – *Macrophomina phaseolina* and *Fusarium thapsinum,* respectively.
