# Supplementary material for: Genome-Wide Association Study on Resistance to Stalk Rot Diseases in Grain Sorghum
Source: G3 (Bethesda). 2015 Apr 16;5(6):1165–75. doi: 10.1534/g3.114.016394 (PMC4478546; doi:10.1534/g3.114.016394)
Supplement: Supporting Information [file supp_g3.114.016394_TableS3.pdf]

Table S3 List of sorghum genotypes included in the population structure analysis (based on 25,000 SNPs) showing the subpopulation assignment and probability values of membership.

Taxa	New assignment	Traditional classification	Caudatum	Bicolor	Kafir	Durra	Guinea	Taxa	New assignment	Traditional classification	Caudatum	Bicolor	Kafir	Durra	Guinea
PI152651	MIXED	cultivar	0.22	0.42	0.18	0.04	0.13	PI576366	MIXED	Durra-Bicolor	0.23	0.32	0.13	0.32	0.01
PI34911	Caudatum	Kafir	0.85	0.15	0.00	0.00	0.00	PI576373	MIXED	Caudatum-B	0.16	0.26	0.48	0.08	0.03
PI533750	Durra	Durra-B	0.00	0.00	0.00	0.94	0.06	PI576375	DURRA	Durra-Bicolor	0.00	0.00	0.06	0.85	0.09
PI533752	Caudatum	Caudatum	0.82	0.07	0.11	0.00	0.00	PI576376	DURRA	Durra-Bicolor	0.00	0.02	0.00	0.97	0.00
PI533754	Mixed	Bicolor	0.03	0.17	0.00	0.53	0.26	PI576380	CAUDATUM	Caudatum	0.98	0.00	0.00	0.00	0.02
PI533755	Mixed	Caudatum	0.22	0.58	0.00	0.10	0.10	PI576381	DURRA	Durra-Bicolor	0.00	0.01	0.02	0.98	0.00
PI533757	Caudatum	Kafir-C	1.00	0.00	0.00	0.00	0.00	PI576385	MIXED	Kafir	0.00	0.00	0.52	0.00	0.48
PI533758	Caudatum	Caudatum	0.99	0.00	0.01	0.00	0.00	PI576386	CAUDATUM	Other	0.85	0.02	0.02	0.07	0.04
PI533759	Caudatum	Caudatum	0.88	0.10	0.00	0.00	0.02	PI576387	CAUDATUM	Other	0.91	0.05	0.00	0.03	0.00
PI533761	Mixed	Durra	0.55	0.36	0.00	0.01	0.08	PI576390	MIXED	Durra	0.00	0.18	0.11	0.62	0.09
PI533762	Mixed	Durra- C	0.48	0.36	0.02	0.03	0.12	PI576391	DURRA	Bicolor	0.00	0.20	0.01	0.80	0.00
PI533766	Guinea	Guinea	0.00	0.02	0.00	0.00	0.98	PI576393	MIXED	Kafir	0.50	0.15	0.12	0.03	0.20
PI533769	Mixed	Kafir-C	0.72	0.18	0.00	0.04	0.06	PI576394	KAFIR	Caudatum	0.00	0.01	0.96	0.01	0.02
PI533776	Guinea	Caudatum	0.00	0.00	0.00	0.00	1.00	PI576399	MIXED	Caudatum	0.26	0.21	0.20	0.00	0.32
PI533785	Guinea	Guinea	0.00	0.00	0.00	0.00	1.00	PI576401	DURRA	Durra	0.00	0.19	0.00	0.79	0.02
PI533788	Mixed	Durra-C	0.34	0.30	0.00	0.35	0.02	PI576418	GUINEA	Guinea	0.00	0.00	0.00	0.00	1.00
PI533789	Mixed	Caudatum	0.48	0.39	0.00	0.05	0.08	PI576422	KAFIR	Kafir	0.00	0.00	1.00	0.00	0.00
PI533792	Caudatum	Caudatum	1.00	0.00	0.00	0.00	0.00	PI576425	DURRA	Durra	0.00	0.02	0.00	0.96	0.01
PI533794	Caudatum	Caudatum	1.00	0.00	0.00	0.00	0.00	PI576426	DURRA	Durra-Bicolor	0.00	0.02	0.04	0.94	0.00
PI533799	Caudatum	Caudatum	1.00	0.00	0.00	0.00	0.00	PI576428	CAUDATUM	Caudatum	0.94	0.00	0.06	0.00	0.00
PI533800	Caudatum	Caudatum	0.84	0.09	0.00	0.07	0.00	PI576435	MIXED	Kafir-Bicolor	0.66	0.16	0.11	0.03	0.04
PI533807	Mixed	Kafir-C	0.77	0.12	0.00	0.00	0.11	PI576437	MIXED	Other	0.34	0.23	0.11	0.13	0.20
PI533810	Durra	Durra	0.00	0.15	0.00	0.85	0.00	PI595699	DURRA	Caudatum	0.00	0.14	0.03	0.83	0.00
PI533814	Durra	Durra	0.00	0.15	0.00	0.83	0.02	PI595702	KAFIR	Kafir-Caudatum	0.00	0.01	0.99	0.00	0.00
PI533821	Mixed	Caudatum	0.18	0.29	0.29	0.00	0.24	PI595714	CAUDATUM	Caudatum	0.80	0.14	0.00	0.00	0.05
PI533822	Mixed	Kafir- C	0.76	0.10	0.06	0.00	0.08	PI595718	MIXED	Kafir-Caudatum	0.58	0.30	0.01	0.08	0.02
PI533824	Mixed	Durra- C	0.10	0.23	0.05	0.42	0.20	PI595720	DURRA	Durra-Bicolor	0.00	0.00	0.00	1.00	0.00
PI533831	Kafir	Kafir- C	0.00	0.00	1.00	0.00	0.00	PI595739	CAUDATUM	Caudatum	0.95	0.00	0.02	0.00	0.03
PI533833	Caudatum	Caudatum-B	0.88	0.10	0.02	0.00	0.00	PI595740	MIXED	Caudatum	0.40	0.11	0.40	0.05	0.04
PI533838	Mixed	Caudatum	0.42	0.08	0.13	0.05	0.32	PI595741	MIXED	Durra	0.00	0.09	0.37	0.00	0.53
PI533841	Mixed	Guinea	0.23	0.06	0.04	0.00	0.67	PI595743	MIXED	Guinea-C	0.10	0.00	0.50	0.12	0.28
PI533842	Durra	Durra	0.00	0.15	0.00	0.85	0.00	PI595744	MIXED	Kafir-Caudatum	0.20	0.36	0.27	0.01	0.15
PI533843	Mixed	Guinea	0.12	0.22	0.31	0.23	0.11	PI595745	MIXED	Guinea-C	0.75	0.11	0.07	0.06	0.00
PI533845	Mixed	Guinea	0.11	0.22	0.48	0.05	0.14	PI597945	MIXED	Durra	0.02	0.27	0.04	0.54	0.13
PI533852	Mixed	Durra	0.00	0.16	0.08	0.75	0.00	PI597946	MIXED	Guinea-Bicolor	0.51	0.08	0.39	0.03	0.00
PI533855	Mixed	Guinea-B	0.06	0.21	0.46	0.15	0.12	PI597950	MIXED	Kafir-Bicolor	0.13	0.24	0.31	0.21	0.11
PI533856	Durra	Durra	0.00	0.15	0.00	0.85	0.00	PI597951	GUINEA	Guinea	0.00	0.01	0.08	0.00	0.91
PI533866	Mixed	Caudatum-B	0.15	0.21	0.02	0.14	0.48	PI597952	MIXED	Caudatum	0.61	0.15	0.17	0.04	0.04
PI533869	Mixed	Guinea	0.10	0.21	0.49	0.00	0.20	PI597957	KAFIR	Durra-Bicolor	0.00	0.00	0.08	0.83	0.09
PI533871	Guinea	Caudatum	0.10	0.04	0.03	0.00	0.83	PI597960	GUINEA	Caudatum-B	0.07	0.04	0.03	0.00	0.86
PI533876	Mixed	Caudatum	0.41	0.26	0.10	0.06	0.17	PI597961	CAUDATUM	Caudatum	0.89	0.01	0.10	0.00	0.00
PI533877	Guinea	Caudatum	0.00	0.00	0.00	0.00	1.00	PI597964	CAUDATUM	Caudatum	0.99	0.00	0.00	0.00	0.01
PI533878	Guinea	Caudatum	0.00	0.00	0.05	0.00	0.95	PI597965	CAUDATUM	Caudatum	0.93	0.07	0.00	0.00	0.00

Taxa	New assignment	Traditional classification	Caudatum	Bicolor	Kafir	Durra	Guinea	Taxa	New assignment	Traditional classification	Caudatum	Bicolor	Kafir	Durra	Guinea
PI533901	Mixed	Caudatum	0.45	0.30	0.11	0.03	0.12	PI597966	CAUDATUM	Caudatum	0.87	0.08	0.05	0.00	0.00
PI533902	Mixed	Durra-B	0.02	0.28	0.05	0.47	0.18	PI597967	CAUDATUM	Caudatum	1.00	0.00	0.00	0.00	0.00
PI533910	Mixed	Caudatum	0.53	0.26	0.17	0.03	0.01	PI597971	MIXED	Caudatum	0.50	0.26	0.11	0.07	0.06
PI533911	Mixed	Caudatum	0.55	0.22	0.01	0.06	0.16	PI597972	MIXED	Durra-Caudatum	0.34	0.25	0.02	0.27	0.11
PI533912	Mixed	Caudatum	0.46	0.34	0.17	0.00	0.03	PI597973	MIXED	Durra-Bicolor	0.12	0.26	0.00	0.43	0.19
PI533913	Mixed	Guinea-C	0.43	0.14	0.19	0.03	0.21	PI597976	GUINEA	Guinea	0.00	0.01	0.00	0.00	0.99
PI533919	Mixed	Durra-B	0.00	0.11	0.00	0.75	0.14	PI597980	MIXED	Caudatum	0.73	0.11	0.00	0.00	0.17
PI533927	Mixed	Bicolor	0.12	0.27	0.33	0.08	0.20	PI597982	MIXED	Caudatum	0.52	0.36	0.07	0.01	0.05
PI533937	Kafir	Kafir	0.00	0.00	1.00	0.00	0.00	PI607931	MIXED	Cultivar	0.52	0.07	0.41	0.00	0.00
PI533938	Mixed	Caudatum	0.10	0.13	0.42	0.15	0.21	PI613536	MIXED	Caudatum-B	0.27	0.21	0.00	0.38	0.14
PI533939	Mixed	Durra	0.28	0.29	0.19	0.07	0.17	PI629034	MIXED	Breedingline	0.55	0.09	0.00	0.00	0.36
PI533940	Kafir	Bicolor	0.00	0.03	0.80	0.14	0.03	PI629040	MIXED	Inbredline	0.27	0.09	0.08	0.33	0.24
PI533943	Mixed	Durra-B	0.00	0.13	0.30	0.55	0.02	PI641874	MIXED	Na	0.00	0.01	0.35	0.64	0.00
PI533948	Kafir	Guinea-Kafir	0.00	0.00	1.00	0.00	0.00	PI655977	CAUDATUM	Breedingline	0.85	0.08	0.00	0.07	0.00
PI533955	Kafir	Kafir-C	0.00	0.00	1.00	0.00	0.00	PI655978	MIXED	Breedingline	0.00	0.15	0.15	0.06	0.64
PI533956	Mixed	Durra-B	0.09	0.12	0.38	0.14	0.28	PI655979	MIXED	Inbredline	0.24	0.20	0.04	0.09	0.44
PI533957	Mixed	Caudatum	0.19	0.17	0.22	0.12	0.30	PI655986	MIXED	Breedingline	0.01	0.11	0.47	0.39	0.03
PI533961	Caudatum	Caudatum	0.87	0.08	0.05	0.00	0.00	PI655987	KAFIR	Na	0.00	0.00	0.92	0.08	0.00
PI533962	Caudatum	Caudatum	0.99	0.01	0.00	0.00	0.00	PI655989	KAFIR	Breedingline	0.00	0.04	0.80	0.14	0.02
PI533964	Caudatum	Caudatum	1.00	0.00	0.00	0.00	0.00	PI655990	MIXED	Kafir	0.01	0.13	0.16	0.66	0.04
PI533965	Caudatum	Caudatum-B	0.80	0.12	0.00	0.00	0.08	PI655991	KAFIR	Kafir	0.00	0.00	1.00	0.00	0.00
PI533967	Caudatum	Caudatum	0.94	0.06	0.00	0.00	0.00	PI655992	KAFIR	Kafir	0.00	0.00	1.00	0.00	0.00
PI533970	Caudatum	Caudatum	0.90	0.08	0.02	0.00	0.00	PI655993	KAFIR	Kafir	0.00	0.00	0.85	0.15	0.00
PI533972	Caudatum	Caudatum	1.00	0.00	0.00	0.00	0.00	PI655996	MIXED	Breedingline	0.14	0.18	0.00	0.00	0.67
PI533976	Caudatum	Caudatum	0.84	0.06	0.09	0.00	0.00	PI655998	MIXED	Na	0.00	0.00	0.33	0.00	0.67
PI533979	Kafir	Kafir	0.00	0.00	1.00	0.00	0.00	PI656001	MIXED	Breedingline	0.64	0.10	0.13	0.14	0.00
PI533985	Caudatum	Caudatum	0.90	0.10	0.00	0.00	0.00	PI656010	GUINEA	Na	0.00	0.14	0.00	0.00	0.86
PI533986	Mixed	Caudatum	0.67	0.29	0.00	0.01	0.02	PI656015	MIXED	Bicolor	0.23	0.58	0.00	0.08	0.10
PI533987	Mixed	Caudatum	0.59	0.32	0.00	0.02	0.07	PI656018	KAFIR	Breedingline	0.00	0.00	0.94	0.06	0.00
PI533989	Caudatum	Durra	1.00	0.00	0.00	0.00	0.00	PI656019	MIXED	Kafir	0.00	0.00	0.69	0.23	0.08
PI533991	Caudatum	Guinea-C	0.80	0.00	0.14	0.06	0.00	PI656022	KAFIR	Breedingline	0.01	0.01	0.99	0.00	0.00
PI533996	Mixed	Durra-C	0.41	0.33	0.05	0.09	0.12	PI656023	KAFIR	Kafir	0.00	0.00	1.00	0.00	0.00
PI533997	Mixed	Guinea	0.08	0.19	0.40	0.07	0.26	PI656025	MIXED	Na	0.00	0.30	0.00	0.66	0.04
PI533998	Kafir	Guinea	0.00	0.04	0.86	0.07	0.04	PI656027	MIXED	Cultivar	0.76	0.09	0.01	0.13	0.02
PI534009	Mixed	Durra	0.00	0.18	0.06	0.63	0.13	PI656029	DURRA	Durra	0.00	0.00	0.11	0.89	0.00
PI534021	Durra	Durra	0.00	0.15	0.00	0.85	0.00	PI656034	CAUDATUM	Cultivar	1.00	0.00	0.00	0.00	0.00
PI534028	Mixed	Durra	0.00	0.13	0.10	0.77	0.00	PI656035	MIXED	Na	0.73	0.23	0.01	0.00	0.04
PI534037	Mixed	Guinea-C	0.05	0.11	0.29	0.04	0.52	PI656048	MIXED	Cultivar	0.73	0.00	0.27	0.00	0.00
PI534053	Mixed	Caudatum	0.39	0.40	0.08	0.10	0.04	PI656051	CAUDATUM	Cultivar	0.91	0.00	0.00	0.00	0.09
PI534054	Mixed	Kafir-C	0.06	0.13	0.69	0.00	0.12	PI656058	MIXED	Kafir	0.37	0.04	0.58	0.00	0.00
PI534063	Guinea	Guinea-C	0.00	0.00	0.00	0.00	1.00	PI656063	MIXED	Na	0.23	0.00	0.57	0.19	0.00
PI534070	Guinea	Guinea	0.00	0.00	0.00	0.00	1.00	PI656071	CAUDATUM	Caudatum	0.80	0.13	0.00	0.04	0.02
PI534075	Guinea	Caudatum	0.00	0.00	0.00	0.00	1.00	PI656072	DURRA	Durra	0.00	0.00	0.00	1.00	0.00
PI534079	Mixed	Caudatum-B	0.52	0.07	0.00	0.04	0.38	PI656074	MIXED	Guinea-C	0.24	0.46	0.07	0.05	0.18
PI534096	Mixed	Guinea	0.07	0.27	0.15	0.10	0.41	PI656075	CAUDATUM	Guinea	0.91	0.00	0.09	0.00	0.00

Taxa	New assignment	Traditional classification	Caudatum	Bicolor	Kafir	Durra	Guinea	Taxa	New assignment	Traditional classification	Caudatum	Bicolor	Kafir	Durra	Guinea
PI534097	Kafir	Kafir	0.00	0.02	0.89	0.09	0.00	PI656076	CAUDATUM	Caudatum	0.97	0.00	0.03	0.00	0.00
PI534099	Caudatum	Caudatum	0.90	0.10	0.00	0.00	0.00	PI656077	MIXED	Durra-Bicolor	0.05	0.12	0.00	0.72	0.11
PI534101	Mixed	Caudatum	0.72	0.13	0.09	0.01	0.04	PI656078	MIXED	Kafir-Durra	0.07	0.28	0.13	0.10	0.42
PI534104	Mixed	Kafir-C	0.53	0.38	0.00	0.04	0.05	PI656080	MIXED	Guinea	0.09	0.18	0.55	0.00	0.17
PI534105	Caudatum	Kafir-C	0.89	0.06	0.05	0.00	0.00	PI656081	MIXED	Guinea	0.07	0.31	0.18	0.09	0.35
PI534108	Caudatum	Kafir-C	0.89	0.00	0.11	0.00	0.00	PI656082	DURRA	Bicolor	0.00	0.10	0.00	0.86	0.04
PI534112	Caudatum	Other	0.98	0.00	0.02	0.00	0.00	PI656083	MIXED	Caudatum	0.48	0.41	0.01	0.00	0.10
PI534114	Caudatum	Caudatum	0.92	0.00	0.08	0.00	0.00	PI656085	CAUDATUM	Na	0.89	0.00	0.11	0.00	0.00
PI534116	Mixed	Durra-B	0.06	0.17	0.22	0.39	0.16	PI656086	MIXED	Durra-Bicolor	0.38	0.21	0.00	0.30	0.11
PI534117	Mixed	Bicolor	0.07	0.26	0.33	0.03	0.31	PI656088	DURRA	Other	0.00	0.04	0.00	0.96	0.00
PI534123	Mixed	Durra-B	0.09	0.29	0.07	0.35	0.21	PI656089	DURRA	Durra	0.00	0.05	0.00	0.95	0.00
PI534124	Mixed	Guinea-B	0.07	0.29	0.06	0.33	0.24	PI656090	MIXED	Guinea-C	0.40	0.29	0.13	0.08	0.10
PI534127	Durra	Kafir-B	0.00	0.08	0.02	0.84	0.06	PI656091	DURRA	Durra	0.00	0.00	0.00	1.00	0.00
PI534128	Durra	Durra	0.00	0.00	0.04	0.96	0.00	PI656092	DURRA	Durra	0.00	0.00	0.05	0.95	0.00
PI534132	Durra	Durra	0.00	0.00	0.07	0.93	0.00	PI656093	GUINEA	Guinea	0.00	0.01	0.08	0.06	0.85
PI534133	Durra	Durra	0.00	0.00	0.00	1.00	0.00	PI656094	GUINEA	Guinea	0.00	0.01	0.00	0.00	0.99
PI534135	Durra	Durra	0.00	0.00	0.00	1.00	0.00	PI656095	GUINEA	Caudatum	0.00	0.07	0.00	0.00	0.93
PI534137	Mixed	Caudatum	0.36	0.30	0.05	0.24	0.05	PI656096	GUINEA	Caudatum	0.00	0.00	0.00	0.00	1.00
PI534138	Mixed	Caudatum	0.24	0.40	0.12	0.01	0.23	PI656097	DURRA	Durra	0.00	0.15	0.00	0.85	0.00
PI534139	Mixed	Guinea-C	0.06	0.24	0.35	0.11	0.24	PI656099	DURRA	Durra	0.00	0.15	0.00	0.85	0.00
PI534144	Mixed	Durra-C	0.15	0.35	0.14	0.02	0.34	PI656100	DURRA	Durra	0.00	0.15	0.00	0.85	0.00
PI534145	Guinea	Other	0.00	0.02	0.00	0.00	0.98	PI656101	MIXED	Guinea	0.28	0.57	0.00	0.06	0.09
PI534148	Durra	Durra-B	0.00	0.03	0.00	0.97	0.00	PI656102	MIXED	Caudatum-B	0.29	0.20	0.26	0.00	0.25
PI534155	Durra	Durra-B	0.01	0.10	0.02	0.80	0.06	PI656103	MIXED	Bicolor	0.00	0.22	0.00	0.66	0.12
PI534157	Caudatum	Caudatum	0.97	0.00	0.00	0.00	0.03	PI656104	MIXED	Bicolor	0.00	0.15	0.24	0.62	0.00
PI534163	Mixed	Caudatum	0.67	0.02	0.31	0.00	0.00	PI656105	MIXED	Kafir-Caudatum	0.69	0.00	0.28	0.03	0.00
PI534167	Mixed	Durra-B	0.00	0.07	0.11	0.72	0.10	PI656106	CAUDATUM	Caudatum	0.87	0.00	0.13	0.00	0.00
PI542718	Durra	NA	0.00	0.30	0.00	0.66	0.04	PI656107	MIXED	Caudatum	0.47	0.39	0.00	0.11	0.02
PI561071	Mixed	Inbredline	0.20	0.19	0.11	0.17	0.33	PI656108	DURRA	Durra	0.00	0.15	0.05	0.80	0.00
PI561472	Caudatum	Cultivar	0.92	0.04	0.04	0.00	0.00	PI656110	MIXED	Durra-Bicolor	0.00	0.21	0.01	0.61	0.17
PI576332	Kafir	Bicolor	0.01	0.08	0.86	0.05	0.01	PI656111	MIXED	Kafir-Durra	0.18	0.16	0.44	0.00	0.23
PI576333	Kafir	Guinea-K	0.00	0.00	1.00	0.00	0.00	PI656112	MIXED	Guinea-Kafir	0.01	0.09	0.22	0.57	0.11
PI576339	Kafir	Kafir-C	0.00	0.00	1.00	0.00	0.00	PI656113	GUINEA	Durra	0.00	0.00	0.00	0.00	1.00
PI576340	Kafir	Kafir-C	0.00	0.00	1.00	0.00	0.00	PI656114	MIXED	Durra-Bicolor	0.21	0.01	0.19	0.43	0.16
PI576345	Kafir	Caudatum	0.00	0.06	0.92	0.00	0.03	PI656115	MIXED	Guinea	0.07	0.27	0.19	0.08	0.38
PI576347	Mixed	Bicolor	0.19	0.30	0.20	0.17	0.14	PI656117	CAUDATUM	Caudatum	1.00	0.00	0.00	0.00	0.00
PI576348	Mixed	Bicolor	0.03	0.30	0.22	0.26	0.19	PI656118	CAUDATUM	Na	0.89	0.08	0.00	0.00	0.03
PI576349	Mixed	Bicolor	0.05	0.32	0.18	0.29	0.16	PI656119	GUINEA	Caudatum	0.00	0.00	0.15	0.00	0.85
PI576350	Mixed	Kafir-C	0.08	0.23	0.35	0.16	0.18	PI656120	MIXED	Caudatum	0.74	0.00	0.22	0.04	0.00
PI576352	Kafir	Kafir	0.00	0.00	1.00	0.00	0.00	PI656121	MIXED	Na	0.44	0.11	0.05	0.08	0.32
PI576364	Kafir	Caudatum	0.00	0.04	0.94	0.02	0.00								

B - Bicolor; C- caudatum; D - Durra; G - Guinea; K - Kafir.
