# Supplementary material for: Genome-Wide Association Study on Resistance to Stalk Rot Diseases in Grain Sorghum
Source: G3 (Bethesda). 2015 Apr 16;5(6):1165–75. doi: 10.1534/g3.114.016394 (PMC4478546; doi:10.1534/g3.114.016394)
Supplement: Supporting Information [file supp_g3.114.016394_TableS4.pdf]

Table S4 Chromosome locations, and other summary statistics for SNPs significantly associated with stalk rot resistance in the combined, Manhattan combined and Ottawa analysis based on the unified mixed model.

Chromosome	SNP Physical Position, bp	P-Value	MAF[a]	Disease traits[b]
Combined analysis				
9	57816733	2.87E-06	0.28	MLL (*FT*)
9	57272115	4.18E-06	0.25	MLL (*FT*)
6	60030948	5.65E-06	0.10	MLL (*FT*)
9	57222599	1.48E-07	0.28	MLL (*MP*)
9	57476134	2.28E-07	0.30	MLL (*MP*)
9	57272296	5.58E-07	0.27	MLL (*MP*)
9	56152890	6.56E-07	0.34	MLL (*MP*)
9	57169768	7.50E-07	0.23	MLL (*MP*)
9	57272115	7.72E-07	0.25	MLL (*MP*)
3	60176979	4.97E-05	0.23	RMLL (*FT*)
2	64623580	9.16E-05	0.44	RMLL (*FT*)
7	58280357	1.43E-05	0.35	RMLL (*MP*)
7	56256841	1.95E-05	0.39	RMLL (*MP*)
3	60176979	7.62E-05	0.23	RTLL (*FT*)
7	56152038	1.35E-05	0.47	RTLL (*MP*)
9	56152890	1.87E-05	0.34	RTLL (*MP*)
9	57272115	1.72E-06	0.25	TLL (*FT*)
9	57222599	2.07E-06	0.28	TLL (*FT*)
9	57816733	2.66E-06	0.28	TLL (*FT*)
9	57222599	8.93E-08	0.28	TLL (*MP*)
9	56152890	1.17E-07	0.34	TLL (*MP*)
9	57476134	2.25E-07	0.30	TLL (*MP*)
9	56508161	4.94E-07	0.41	TLL (*MP*)
9	57236778	5.82E-07	0.29	TLL (*MP*)
9	57272296	6.91E-07	0.27	TLL (*MP*)
9	57272115	7.47E-07	0.25	TLL (*MP*)
9	56562984	9.71E-07	0.34	TLL (*MP*)
Manhattan combined analysis				
9	57272115	2.93E-06	0.25	MLL (*FT*)
9	57816733	3.36E-06	0.28	MLL (*FT*)
9	57383556	6.39E-06	0.30	MLL (*FT*)
9	57236791	6.70E-06	0.30	MLL (*FT*)
9	57272115	4.04E-07	0.25	MLL (*MP*)
9	57272296	6.16E-07	0.27	MLL (*MP*)
9	57476134	6.53E-07	0.30	MLL (*MP*)
9	57169768	6.69E-07	0.23	MLL (*MP*)
9	57383556	9.89E-07	0.30	MLL (*MP*)
2	60129082	9.67E-06	0.40	RMLL (*MP*)

Chromosome	SNP Physical Position, bp	P-Value	MAF[a]	Disease traits[b]
6	59739236	1.89E-05	0.46	RTLL (*FT*)
2	64623580	1.61E-05	0.44	RMLL (*FT*)
7	59890439	1.07E-05	0.32	RTLL (*MP*)
9	57272115	2.48E-06	0.25	TLL (*FT*)
9	57816733	5.56E-06	0.28	TLL (*FT*)
9	57236791	6.72E-06	0.30	TLL (*FT*)
9	56152890	2.82E-07	0.34	TLL (*MP*)
9	57272115	4.34E-07	0.25	TLL (*MP*)
9	57383556	4.99E-07	0.30	TLL (*MP*)
9	57222599	5.93E-07	0.28	TLL (*MP*)
9	57476134	9.28E-07	0.30	TLL (*MP*)
Ottawa analysis				
1	11814753	1.70E-05	0.12	MLL (*FT*)
4	55646941	1.78E-05	0.31	MLL (*FT*)
2	69967056	3.73E-05	0.17	MLL (*MP*)
1	42155829	5.19E-05	0.10	MLL (*MP*)
3	60195553	4.48E-05	0.31	RMLL (*FT*)
4	55646941	6.59E-05	0.31	RMLL (*FT*)
1	66836006	1.20E-05	0.08	RMLL (*MP*)
8	51279094	2.55E-05	0.40	RMLL (*MP*)
6	54221186	6.27E-05	0.17	RTLL (*FT*)
10	3137796	8.26E-05	0.17	RTLL (*FT*)
1	61119284	2.68E-05	0.32	RTLL (*MP*)
8	51279094	3.48E-05	0.40	RTLL (*MP*)
10	3137796	2.38E-05	0.17	RTLL (*FT*)
9	56802949	2.61E-05	0.18	RTLL (*FT*)
1	27040589	6.39E-05	0.32	TTL (*MP*)
1	61119285	6.57E-05	0.32	TTL (*MP*)

[a]MAF, minor allele frequency.

[b]Disease traits: TLL and MLL – total lesion length and major lesion length, respectively; RTLL and RMLL – relative total lesion length and relative major lesion length, respectively; *MP* and *FT* – *Macrophomina phaseolina* and *Fusarium thapsinum,* respectively.
