# Supplementary material for: Genome-Wide Association Study on Resistance to Stalk Rot Diseases in Grain Sorghum
Source: G3 (Bethesda). 2015 Apr 16;5(6):1165–75. doi: 10.1534/g3.114.016394 (PMC4478546; doi:10.1534/g3.114.016394)
Supplement: Supporting Information [file supp_g3.114.016394_FigureS3.pdf]

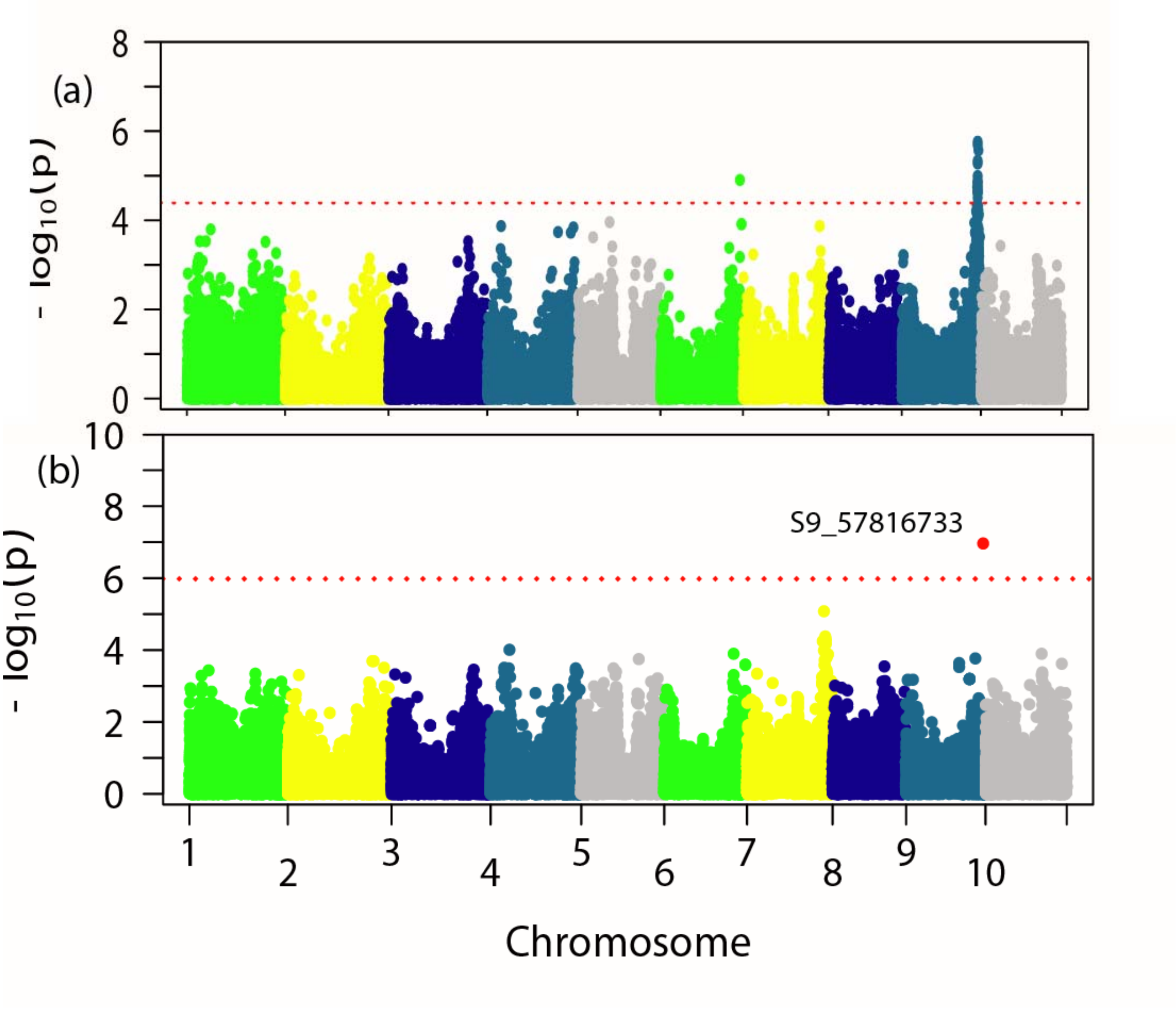

Figure S3 Plots of SNPs associated with major lesion length (TLL) for *Fusarium thapsinum* based on the combined analysis. (a) Single-locus mixed model (marked in red; Bonferroni-corrected threshold of 0.05; dashed horizontal line); (b) Multi-locus mixed model (MLMM) identifies one SNP on chromosome 9 (marked in red) from the optimum model. The vertical axis indicates the $-\log_{10}$ of P-value scores, and the horizontal axis indicates chromosomes and physical positions of SNPs.
