# Supplementary material for: Genome-Wide Association Study on Resistance to Stalk Rot Diseases in Grain Sorghum
Source: G3 (Bethesda). 2015 Apr 16;5(6):1165–75. doi: 10.1534/g3.114.016394 (PMC4478546; doi:10.1534/g3.114.016394)
Supplement: Supporting Information [file supp_g3.114.016394_FigureS7.pdf]

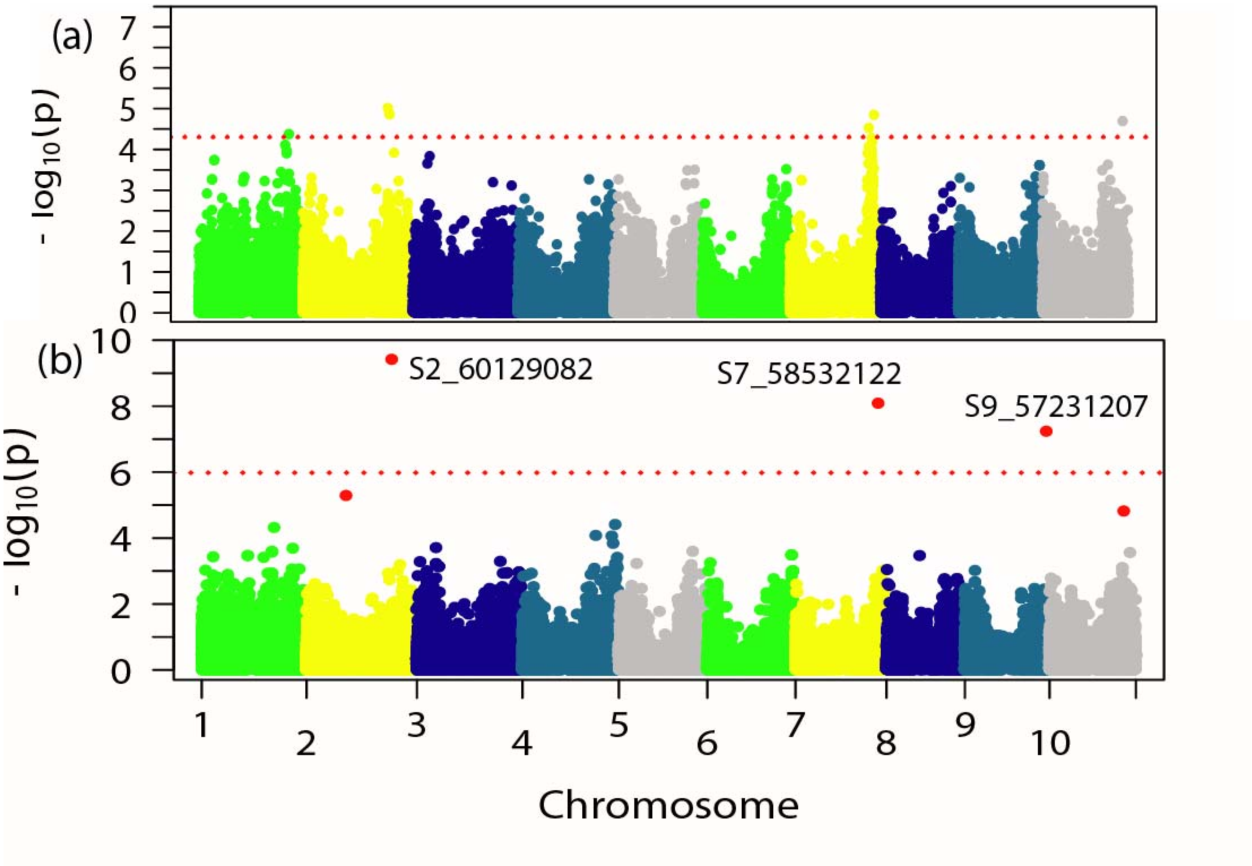

Figure S7 Plots of SNPs associated with relative major lesion length (RMLL) for *Macrphomina phaseolina* based on across year combined data for Manhattan locations. (a) Single-locus mixed model (marked in red; Bonferroni-corrected threshold of 0.05; dashed horizontal line); (b) Multi-locus mixed model (MLMM) which identifies three SNPs on chromosomes 2, 7 and 9 (marked in red) from the optimum model (b). The vertical axis indicates the –$\log_{10}$ of P-value scores, and the horizontal axis indicates chromosomes and physical positions of SNPs.
