# Supplementary material for: Genome-Wide Association Study on Resistance to Stalk Rot Diseases in Grain Sorghum
Source: G3 (Bethesda). 2015 Apr 16;5(6):1165–75. doi: 10.1534/g3.114.016394 (PMC4478546; doi:10.1534/g3.114.016394)
Supplement: Supporting Information [file supp_g3.114.016394_FigureS12.pdf]

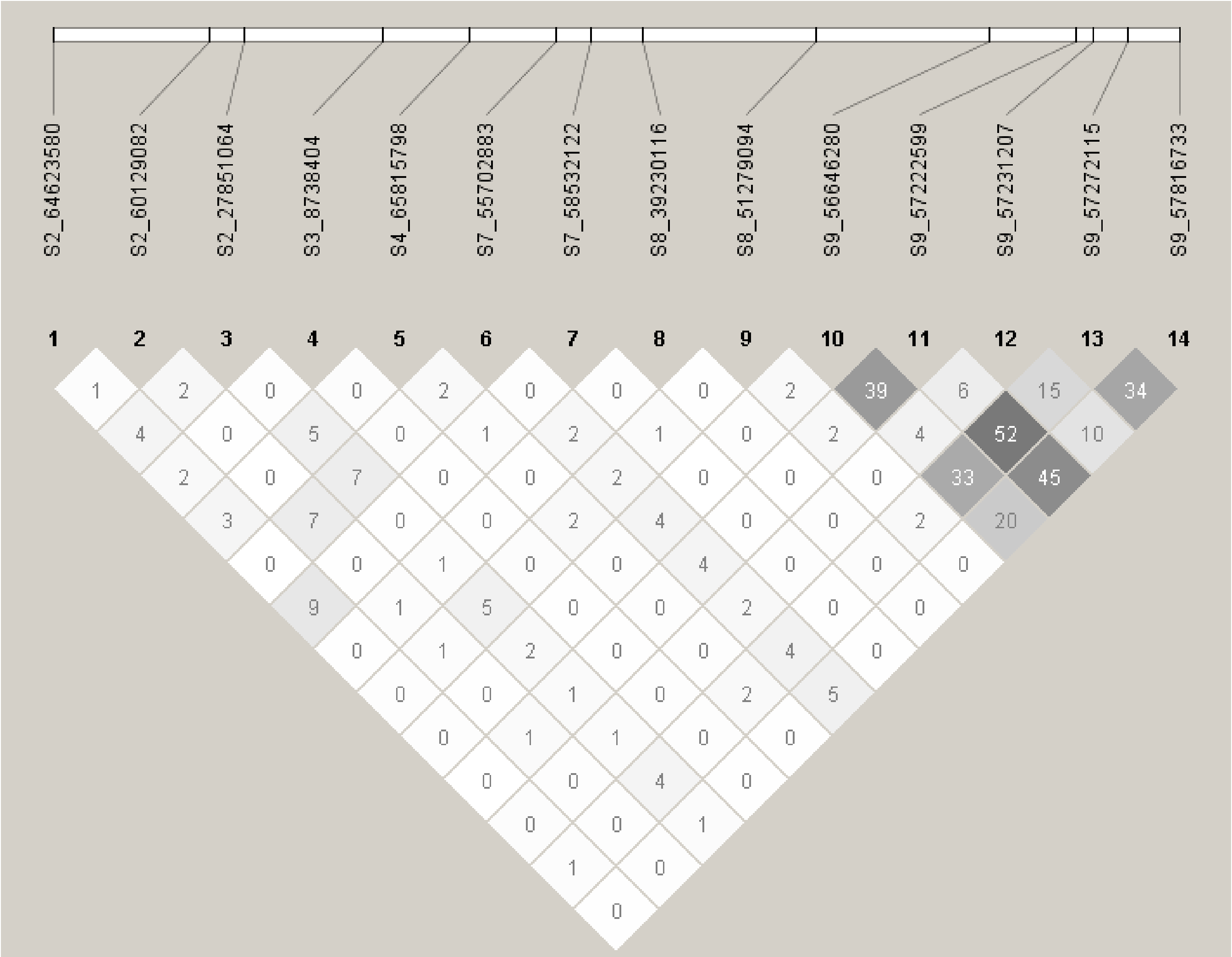

Figure S12 Linkage Disequilibrium (LD) among significant SNP markers. HAPLOVIEW v.4.2 (Barrett et al., 2005) pairwise LD values (r2*100) for 14 SNPs tested based on 257 genotypes to determine whether all the SNPs are significantly associated with stalk rot resistance were in strong LD with each other; white, $r^2=0$; shades of gray, $0 < r^2 < 1$.
